# DNA-programmed bispecific peptide assemblies for delivering cytotoxic payload to cells expressing EGFR and MET receptors

**DOI:** 10.1039/d5cb00238a

**Published:** 2025-12-03

**Authors:** Pritam Ghosh, Huyen Dinh, Oliver Seitz

**Affiliations:** a Institute of Chemistry, Humboldt-Universität zu Berlin Brook-Taylor-Str. 2 D-12489 Berlin Germany oliver.seitz@chemie.hu-berlin.de; b Tam Anh Research Institute 2B Pho Quang, Ward 2 Tan Binh District Hochiminh city 70000 Vietnam

## Abstract

Bispecific agents capable of simultaneously targeting two distinct cell surface receptors promise enhanced specificity and efficacy in cancer therapy. Here, we report a strategy for the rapid optimization of compact bispecific agents using nucleic acid hybridization to display peptide ligands for both the epidermal growth factor receptor (EGFR) and the mesenchymal-epithelial transition factor (MET). The self-assembly process involved 20 and 21 nucleotide (nt) long DNA-peptide conjugates and 41–46 nt template strands, which precisely controlled the spatial arrangement of the EGFR-targeting peptide GE11 and the MET-binding bicyclic peptide GE137. We introduce improved synthetic methods for the challenging construction and functionalization of GE137, enabling its efficient conjugation to oligonucleotides. Systematic variation of peptide spacing revealed a striking distance-dependent affinity profile in interactions with live A549 cells, with optimal staining observed when GE11 and GE137 were separated by 21 paired and 3 unpaired DNA nucleotides. Incorporation of a cleavable cytotoxic payload (monomethyl auristatin E) into bispecific DNA–peptide constructs led to potent, HGF-dependent cytotoxicity, underscoring the requirement for targeted internalization. Conjugation to DNA effectively masked the cytotoxic payload, unless the combined activity of GE11 and GE137 induced internalization. This work establishes that DNA-directed assembly allows precise optimization of bispecific peptide agents that are much smaller than conventional constructs, offering robust targeting and conditional cytotoxicity. These findings highlight the promise of nucleic acid scaffolds for next-generation, cell-selective therapeutics.

## Abstract 2

Interactions of bispecific DNA–peptide complexes with EGFR and MET on A549 cells showed a marked distance-affinity profile. The conditional cytotoxicity of constructs armed with auristatins is contingent on both targeting and cell internalisation.

## Graphical abstract

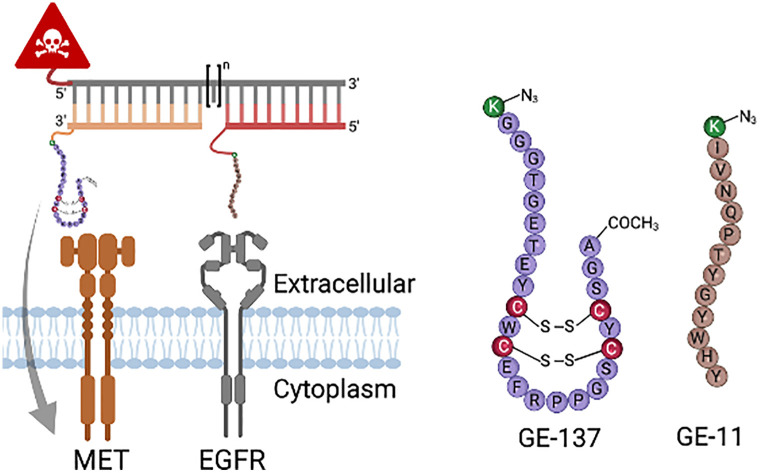

## Introduction

The development of bispecific agents - molecules designed to simultaneously bind two different cell surface receptors – is an avenue promising increased efficacy and specificity in targeted therapies.^1–8^ Most bispecific agents act in *trans*, in order to, for example, recruit immune cells to cancer cells. According to an alternative approach, bispecific molecules are envisaged to bind two cell surface proteins expressed on the same cell.^9,10^ Such molecules could, in principle, harness synergy and, due to bivalency-enhanced binding, exhibit increased affinity for targeted cells.

Among the promising targets are the epidermal growth factor receptor (EGFR) and the mesenchymal-epithelial transition factor (MET), both of which are frequently overexpressed or aberrantly activated in a range of cancers, including lung, breast, gastric, and glioblastoma.^11,12^ These receptors play critical roles in cell proliferation, survival, and metastasis. EGFR and MET are commonly found at high levels in lung and breast cancer. It is believed that co-targeting EGFR and MET with a single bispecific agent could improve therapeutic efficacy in the treatment of non-small cell lung cancer (NSCLC).^13–17^

Traditional bispecific constructs often rely on antibodies, nanobodies, or aptamers as recognition elements. However, peptides offer several key advantages that could make them attractive alternatives. Their small size (∼1–5 kDa) typically allows for improved tumor tissue penetration compared to larger antibody-based molecules. Peptides are also synthetically accessible, enabling cost-effective, scalable production and chemical versatility for functionalization. Peptides can be engineered or selected for high specificity and affinity to EGFR^18–23^ and MET.^24–26^ However, the choice of the linker connecting the two different peptide ligands is not straightforward. Flexible organic tethers, such as bifunctional oligoethyleneglycol derivatives, offer synthetic ease, but the two appended ligands would sample a large distance space and pronounced affinity enhancements can, therefore, only be obtained when distances between binding sites are small.^27,28^

**Fig. 1:**
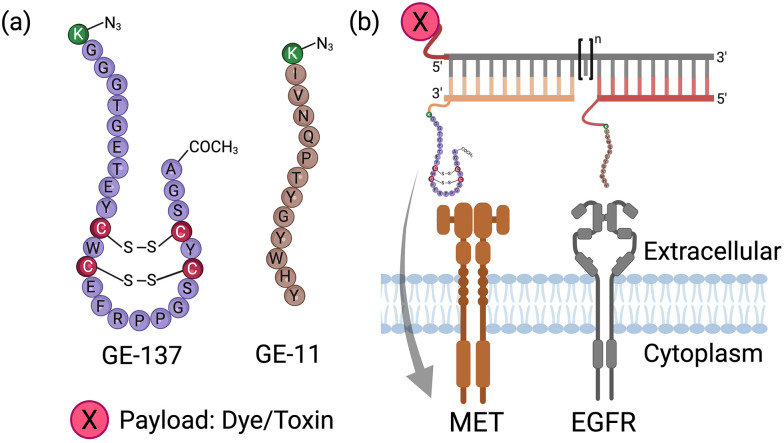
(a) Sequence of peptides GE137 and GE11 used for binding MET and EGFR receptors, respectively. Azido-lysine residues highlighted in green have been added to allow conjugation with oligonucleotides *via* copper-click reactions. (b) Principle of self-assembly to rapidly screen for bispecific DNA–peptide complexes that enable effective targeted delivery of payload into cells expressing both EGFR and MET receptors (pictures made with Biorender.com).

Herein, we explore an alternative approach to constructing bispecific peptide-based agents, which involves the use of DNA as a ruler-like molecular linker (Fig. 1).^27–45^ Short, synthetic DNA strands can serve as programmable, biocompatible scaffolds to spatially organize and connect peptide ligands targeting EGFR and MET.^46^ The nanometer-scale control over the distance and orientation between the two peptides presented on self-assembled complexes enables a convenient optimization for cooperative binding to receptor pairs on the cancer cell surface. The strategy relies on the 12 amino acid long peptide GE11^19,20^ for targeting of EGFR and the 26 amino acid bicyclic peptide GE137^25^ for targeting of MET (Fig. 1). Herein, we provide full experimental details for the challenging synthesis of GE137 and its conjugation to oligonucleotides. We demonstrate that, beyond targeting capabilities, bispecific DNA–peptide agents can also serve as platforms for payload delivery, enabling targeted cytotoxicity against cancer cells expressing EGFR and MET receptors.

## Results and discussion

### Synthesis of peptides

The EGFR has long been recognized as a target for cancer therapies. As an alternative to antibodies, peptides have been developed for EGFR targeting. The 12-mer GE11^19,20^ is a widely used EGFR binder and was therefore selected as a recognition module in bispecific DNA–peptide complexes (Fig. 1a). For targeting of the MET receptor, we selected the 26-amino-acid peptide GE137 (Fig. 1a), which has been used for real-time tumor imaging of MET expression during surgery and endoscopy.^25^

**Fig. 2:**
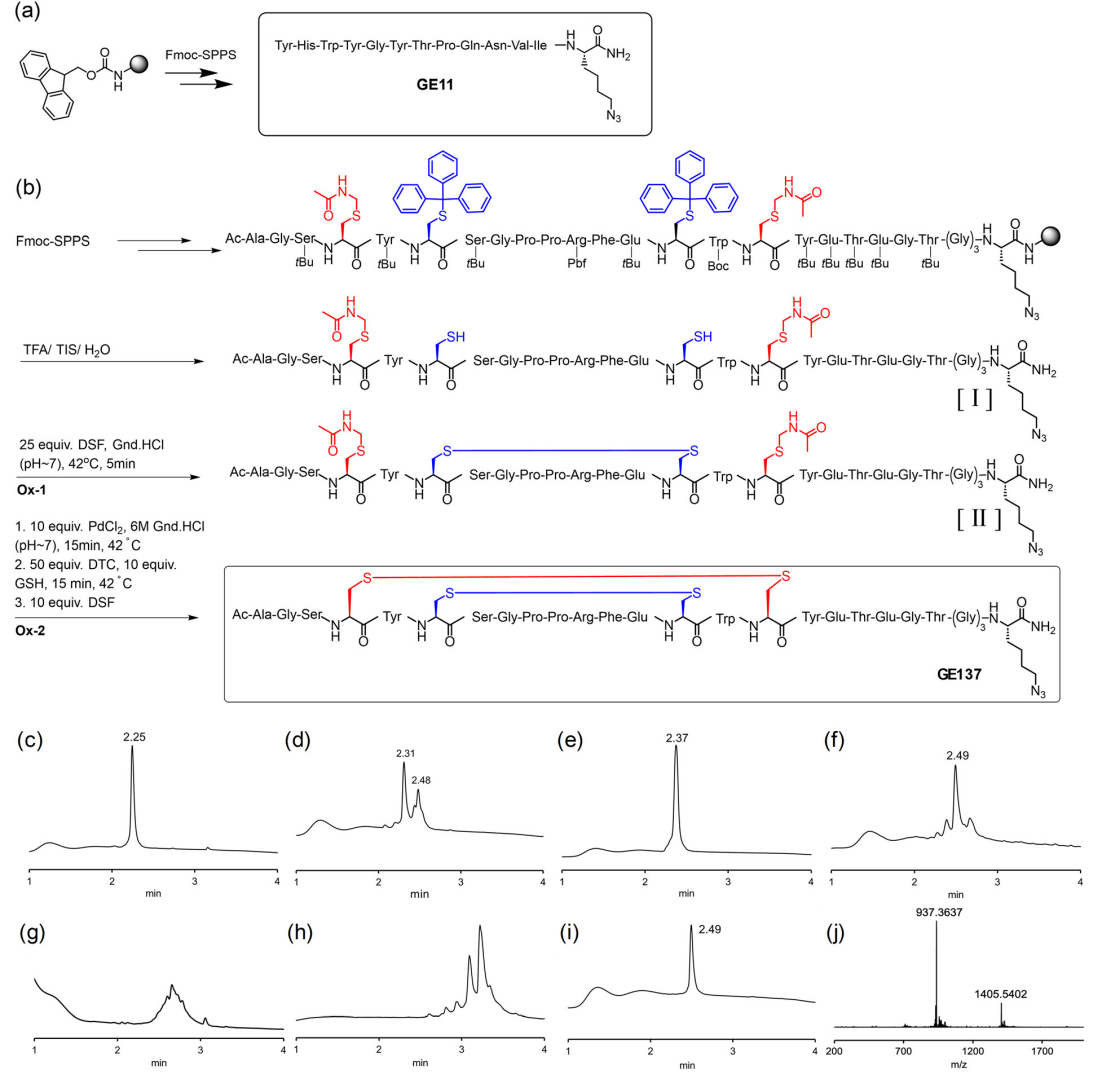
Synthesis of (a) azido-modified GE11 and (b) azido-modified GE137. UPLC analysis of (c) peptide [I] after HPLC purification; (d) mixture formed upon attempting Brik's one pot two disulfide bond formation; (e) peptide [II] after step Ox-1 and HPLC purification; (f) crude GE137 after Ox-2 using GSH; (g) mixture obtained upon attempting Ox-2 using the I_2_/MeOH method; (h) mixture after attempting Ox-2 using the AgOTf method; (i) GE137 after HPLC purification; condition: at *λ* = 210 nm (3–80% solvent B in 6 min), mobile phases: A (98.9% H_2_O, 1% ACN, 0.1% TFA) and B (98.9% ACN, 1% H_2_O, 0.1% TFA). (j) HRMS analysis of HPLC purified GE137 (Fig. S5 for the full range).

To enable conjugation with alkyne-modified oligonucleotides both peptides were equipped with an azidolysine residue. The synthesis of the linear peptide GE11 proved straightforward (Fig. 2a). Solid-phase synthesis afforded the conjugation-ready GE11 derivative in 53% overall yield after preparative HPLC purification.

In comparison, the synthesis of GE137 was not a trivial process. This peptide incorporates two disulfide bridges, which need to be formed without damaging the azido group. The solid-phase synthesis was commenced with loading Fmoc-azidolysine [Fmoc-Lys(N_3_)] onto the Tentagel-R-RAM resin (Fig. 2b). Automated SPPS involved the use of Fmoc-protected amino acids dissolved in a solution of NMP (*N*-methyl-2-pyrrolidone) containing OxymaPure (ethyl cyano(hydroxyimino)acetate). For activation, *O*-(6-chlorobenzotriazol-1-yl)-*N*,*N*,*N*′,*N*′-tetramethyluronium hexafluorophosphate (HCTU) and *N*-methylmorpholine (NMM) were added. To enable a selective formation of disulfide bridges post resin detachment, a combination of cysteine-S-trityl and -S-Acm (acetamidomethyl) protection was used. The N-terminally acetylated crude peptide [I] obtained after TFA (trifluoroacetic acid) cleavage was purified by reverse-phase high-performance liquid chromatography (RP-HPLC) (Fig. 2c) and, subsequently, used to form the disulfide bridges. We intended to apply Brik's method^47,48^ of regioselective disulfide bond formation of peptides in one-pot (Ox-1 and Ox-2 in Fig. 2b). For this purpose, the Acm-protected peptide [I] was dissolved in degassed 6 M guanidine hydrochloride (Gn·HCl) at pH 7 and treated with disulfiram (diethyl[(diethylcarbamothioyl)disulfanyl]carbothioamide, DSF). The reaction mixture was kept for 5 min at 42 °C to form the first disulfide bond. Subsequently, PdCl_2_ was added to the reaction mixture to remove the two Acm protecting groups, followed by the addition of the Pd scavenger sodium diethyldithiocarbamate (DTC), and DSF to allow the formation of the next disulfide bond. However, careful inspection of the MS data for the UPLC-MS analysis (Fig. 2d) did not indicate the formation of the desired peptide with two disulfide bridges, and we chose to refrain from attempting HPLC purification. We speculated that the overall process would be more efficient if by-products could be removed once the first disulfide bond had formed. HPLC purification provided the Acm-protected first disulfide-cyclized peptide [II] (Fig. 2e). However, the process involving Pd-promoted Acm removal and subsequent disulfide formation still remained inefficient using the same protocol (data not shown). We hypothesized that the problem could be related to inefficient Pd scavenging in the presence of the first disulfide. After the PdCl_2_-promoted Acm removal, we applied DTC in large excess (50 eq.) and supplemented with 10 eq. of glutathione (GSH) to prevent its oxidation. With this modified protocol, the DSF-induced formation of the second disulfide proceeded smoothly (Fig. 2f) within 15 minutes at 42 °C, generating GE137 in a 24% isolated yield and sufficient purity (Fig. 2i, j). Interestingly, GE137 was not formed in the absence of GSH.^48^ With this slight variation, we applied the Brik method^48^ in a one-pot procedure by skipping HPLC purification after step Ox-1. While highly convenient, this method provided lower yields (11% for one pot *vs.* 24%).

In attempts to increase the yields of azido-functionalized GE137, we considered alternative methods for Acm removal and formation of the second disulfide bridge. According to a frequently applied procedure,^49^ the second-step oxidation was attempted by using iodine in an acidic solution. In the event, 5 mM iodine in methanol (I_2_/MeOH) was added dropwise to a solution of Cys(Acm)-protected GE-137 peptide at a concentration of 0.5 mg mL^−1^ in 0.1 M citric acid until a consistent yellow coloration was achieved. UPLC analysis of the reaction mixture formed upon quenching with ascorbate revealed a heterogeneous composition of products (Fig. 2g). It is known that side reactions are often associated with this reaction, including, for example, the iodination of sensitive residues such as Tyr and Trp that are present in the sequence.^50,51^ Reactions involving silver trifluoromethanesulfonate (AgOTf) followed by treatment with a solution of aqueous HCl in DMSO have been reported to convert S-Acm-protected peptides into disulfide-cyclized products without significant side reactions at oxidation-sensitive amino acids.^52^ However, UPLC-MS analysis of the mixture obtained after removal of the AgCl precipitate did not indicate the formation of the desired product (Fig. 2h). In conclusion, by comparing three different strategies, we identified a modified version of Brik's method – involving Pd-mediated Acm removal and DSF-promoted disulfide formation – as the most suitable for providing sufficient amounts of azido-functionalized GE137 for conjugation with oligonucleotides.

Given the synthetic challenges, it was deemed essential to verify the ability of GE137 to bind MET on cells. The peptide was conjugated with a TAMRA dye (5-carboxytetramethylrhodamine) (Fig. S7), and binding to a MET-expressing cell line was assessed by flow cytometry. Following a brief incubation (10 min) with fluorescently labelled peptide at a concentration of 200 nM in PBS at 37 °C, A549 cells were successfully stained in the TAMRA channel (Fig. S11). In a control experiment, A549 cells were treated with unlabeled peptide first (Fig. S11). After medium exchange, the TAMRA-labelled peptide was added. As anticipated for a specific interaction, the TAMRA signal was weaker in this case compared to staining in the absence of a non-fluorescent competitor.

### Peptide conjugation with DNA strands *via* copper click reaction

**Fig. 3:**
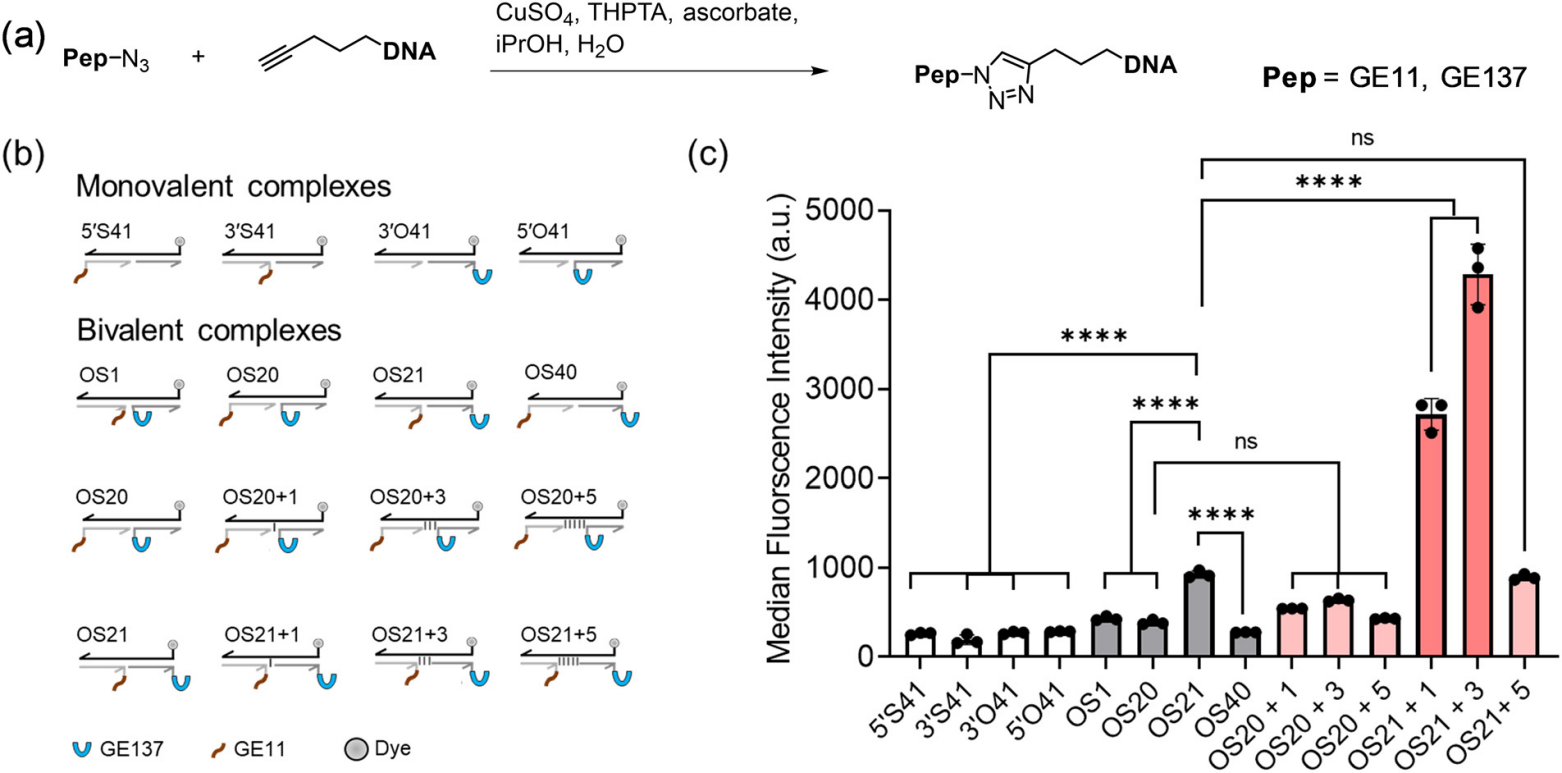
(a) Conjugation GE11-N_3_ and GE137-N_3_ with alkyne-modified oligonucleotides. (b) Library of dsDNA–peptide complexes used in this study. (c) Staining of A549 cells with monovalent and heterobivalent DNA–peptide complexes assessed by flow cytometry of live cells. Conditions: 750 nM, 10 min incubation in PBS at 37 °C, 5% CO_2_.

As previously outlined,^46^ the GE11 and GE137 peptides were linked to 20 and 21-nucleotide-long DNA strands, offering terminal alkyne modifications (for structural details, see chapter F of the SI) suitable for copper click chemistry (Fig. 3a). A collection of self-assembled DNA–peptide complexes (Fig. 3b) was created using a total of 14 different fluorescence-labelled template strands. The four different DNA–peptide conjugates were assembled into 10 bispecific and 4 monovalent complexes (for structural details, see Table S1). The peptides were arranged in a seamless base pairing configuration, with distances of 1 nt, 20 nt, 21 nt, and 40 nt. Furthermore, unpaired spacer nucleotides were incorporated between the duplex segments of the (20 + *n*) and (21 + *n*) complexes. According to a previous investigation, each unpaired nucleotide enhances scaffold flexibility and increases the length of the complexes by 1.2 Å.^29^

### DNA–peptide duplex for staining and delivering a toxic payload to A549 cells

The DNA–peptide complexes were tested for their ability to stain A549 cells, which express both EGFR and MET receptors. The flow cytometric evaluation (Fig. S12 and S13) of staining intensity exposed a remarkably pronounced distance-intensity profile (Fig. 3c). It was observed that monovalent complexes exhibited comparatively weak staining intensity. In the group of bispecific probes (OS1, OS20, OS21, OS40) assembled through seamless base pairing, OS21 produced the highest staining intensity. However, substantial enhancements of staining intensity were observed when three unpaired nucleotides were incorporated between the duplex segments of OS21. Concentration-dependent flow cytometry (Fig. S14) confirmed that OS21+3 exhibited a higher affinity for A549 cells than OS21. When applied at a concentration of 750 nM, the bispecific agent OS21+3 was found to stain A549 cells with an intensity that was approximately 14 times higher than that of monovalent complexes (Fig. S12 and S13).

Two further observations are worthy of note. Firstly, unpaired nucleotides had little effect when incorporated into complex OS20. Secondly, the introduction of five unpaired nucleotides instead of three resulted in a significant decrease in affinity (compare OS21+5 with OS21+3). Complexes OS20 and OS21 differ not only by one base pair in length but also by the position of the appended peptides. In OS20, GE11 is linked to the 5′-position at the duplex end, whereas GE137 stands at the 5′-position of the nick site. In OS21, the positions are reversed (Table S1 for dsDNA complex structure). Given the lower affinity of OS20, it is conceivable that recognition of GE137 is impeded by steric hindrance. However, if steric hindrance were to govern the binding properties, then OS40 should bind well too, since both OS21 and OS40 have GE137 at the less hindered duplex end. This was not observed, which indicates that the distance between the two peptides must play a role. Furthermore, unpaired nucleotides in OS20+1, OS20+3, and OS20+5 are expected to progressively ease steric encumbrance at GE137. However, neither of the complexes demonstrated high affinity. According to an alternative argument, issues related to helicity could impede the process of heterobivalent recognition. Assuming a B-type duplex conformation, the peptides in OS20 would be presented with a helical offset of 34° (34.6° twist per base pair). In OS21, this offset should be close to zero. However, it is important to note that each unpaired nucleotide should increase the ability to undergo torsions around the helix axis. Therefore, if the helical offset were to govern binding, then the OS20+*n* series should exhibit higher binding affinity than was observed. Together with the comparatively weaker binding provided by OS21+5, this suggests that flexibility must not be too high, perhaps, to avoid an entropic penalty. These considerations indicate that a subtle balance between rigidity and flexibility is necessary to achieve heterobivalency-enhanced interactions. The data demonstrate that this can be easily screened using DNA-based scaffolds.

**Fig. 4:**
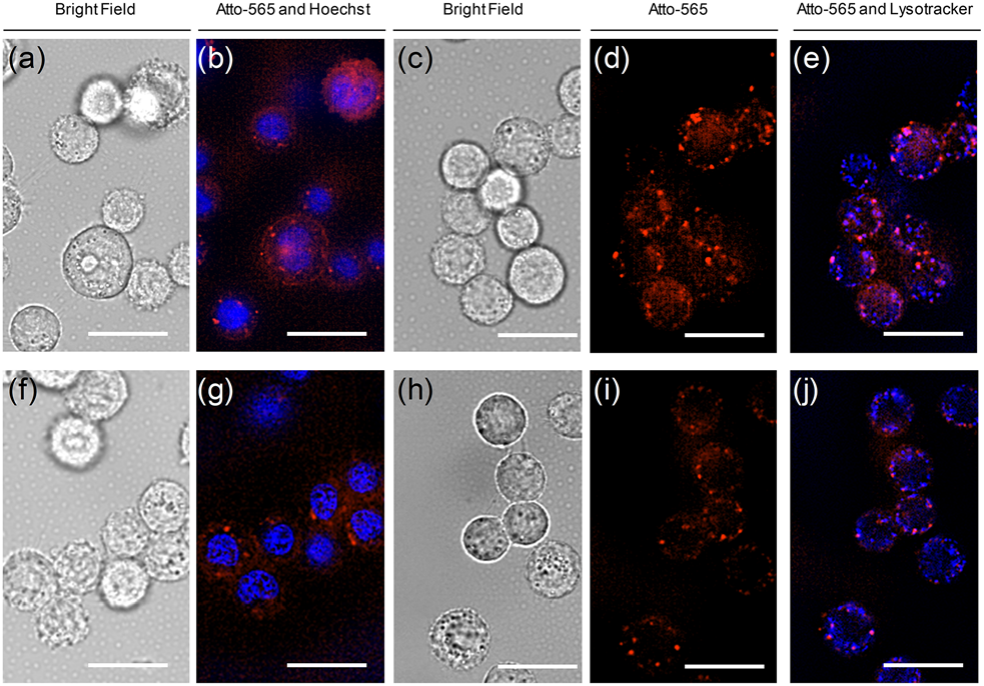
Microscopy images of A549 cells after staining with (a)–(e) OS-21+3 and (f)–(j) OS-21. Red colour is from OS21 or OS21+3. Co-staining was performed with Hoechst33342 (blue colour in (b) and (g)) or Lysotracker (blue colour in (e) and (j)) to localize nuclei or lysosomes, respectively. The purple colour in (e) and (j) indicates localization of OS21+3 or OS21 in lysosomes. Conditions: 1 µM probe in PBS, 30 minutes incubation time, 37 °C, 5% CO_2_; medium: PBS, 50.000 cells seeded per well. Blue: *λ*_ex_ = 350 ± 50 nm, *λ*_em_ = 460 ± 50 nm; red: *λ*_ex_ = 575 ± 25 nm, *λ*_em_ > 593 nm. Full scale figure is shown in Fig. S16. Scale bar is 5 µm.

Targeting molecules have frequently been used to deliver cytotoxic payloads to cancer cells. Typically, binding of the targeting unit to the cell surface receptor triggers receptor-mediated endocytosis. Cleavable linkers are often utilized to enable the release of the cargo within the cells.^53,54^ Targeting molecules that do not induce uptake can still allow delivery of the payload. This requires that the linker can be cleaved by the action of a cancer cell's microenvironment, resulting in the enrichment of the cytotoxic agent at the cancer site. It is our understanding that both delivery routes are, in principle, feasible with the bispecific agents such as OS21 and OS21+3. Of the two targeting ligands used, GE11 triggers internalization of EGFR, while GE137 does not induce internalization of MET.^20,55^ Indeed, fluorescence microscopy analysis of A549 cells treated with OS21 showed predominant cell surface staining and negligible internalization (Fig. S15). However, the internalization of MET can be induced by co-treatment with hepatocyte growth factor (HGF) or epidermal growth factor (EGF), which are present in cancer tissue.^54^ Fluorescence microscopy of A549 cells treated with OS-21 or OS-21+3 and co-stained with Lysotracker suggests that both probes are internalized *via* receptor-mediated endocytosis (Fig. 4).

**Fig. 5:**
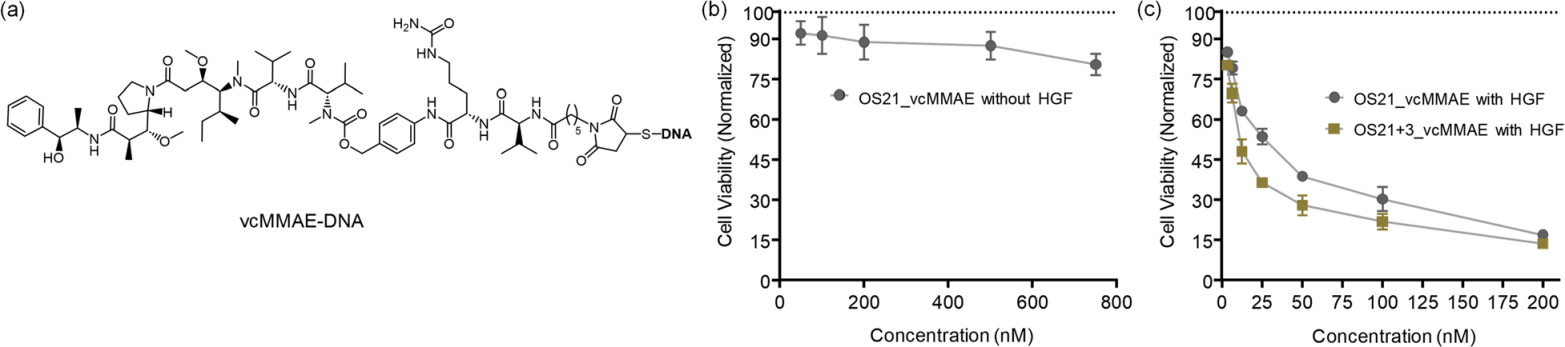
(a) Arming DNA-template strands in OS21 and OS21+3 with the toxin vcMMAE. (b), (c) Dose-cytotoxicity response for treatment of A549 cells with cathepsin B-cleavable vcMMAE-DNA–peptide complexes OS21_vcMMAE and OS21+3_vcMMAE in (b) absence and (c) presence of HGF (100 ng mL^−1^) assessed by the AlamarBlue assay. Viability was normalized to PBS treatment (100% value) and ethanol treatment (0% value). Conditions: 10^4^ cells per well in 100 µL medium and propagated 24 h to a confluency of *ca.* 85% cells, probes were incubated for 10 min in PBS, 37 °C, 5% CO_2_; AlamarBlue assay in full medium, 6 h.

The ability to control internalization by an external stimulus allowed us to distinguish between the two possible paths of payload delivery. Bispecific DNA–peptide probes providing high staining efficiency were repurposed for carrying the tubulin polymerization inhibitor monomethyl auristatin E (MMAE) as a cytotoxic payload. A thiolated template strand was conjugated with vcMMAE *via* maleimide chemistry (Fig. 5a, see Chapter G of the SI for structural details). The resulting thiol-maleimide-linked vcMMAE-DNA conjugates were then annealed with shorter DNA strands containing GE137 or GE11 to generate the bispecific agents OS21_vcMMAE and OS21+3_vcMMAE. A lysosomal protease cleavable dipeptide, valine-citrulline, was inserted between the DNA and MMAE to facilitate the release of MMAE upon cleavage by cathepsin B. DNA is vulnerable to nuclease. If nuclease cleavage occurred in the cell medium, then there was a possibility that MMAE could diffuse, albeit poorly, into the cells. In such cases, the difference between treatments in the presence and absence of HGF would be minimal. However, if the DNA scaffold survived the cell's exterior, then a cytotoxic activity should be contingent on internalization of the bispecific DNA–peptide construct.

A549 cells were incubated with the oligonucleotide assemblies for ten minutes. Cell viability was assessed six hours later using an AlamarBlue assay. Following treatment with OS-21_vcMMAE in the absence of HGF, cell viability remained high even at concentrations up to 750 nM (Fig. 5b). For comparison, we assessed the viability response to treatment with MMAE at a 100 nM concentration in unconjugated form. While virtually no cytotoxicity was observed for the MMAE linked to the DNA–peptide complex in OS21_vcMMAE at 100 nM concentration (Fig. 5b), viability was reduced to 79% upon treatment with free MMAE (Fig. S17). This suggests that conjugation with DNA reduces the cytotoxicity of the auristatin MMAE, probably by hindering cell uptake. The experiment also suggests that the DNA assembly was not significantly degraded in the cell medium or on the cells. Next, the cells were treated in the presence of HGF. A clear dose–response was observed (Fig. 5c), with an EC_50_ = 27 nM for treatment with OS21_vcMMAE. As anticipated from concentration-dependent staining experiments (Fig. S14), OS21+3_vcMMAE exhibited even higher toxicity (EC_50_ = 9 nM) towards the A549 cells. Control experiments showed that cell viability was unaffected when cells were treated with HGF alone or with a DNA–peptide assembly lacking the cytotoxic agent vcMMAE (entry 6, Fig. S17). This indicates that the simultaneous targeting of MET and EGFR alone is insufficient to induce a cytotoxic effect. To assess the efficiency of targeted delivery, we compared the cytotoxicity of OS21+3_vcMMAE with that of a DNA-vcMMAE complex lacking the peptide recognition modules. At 100 nM concentration of complexes in the presence of HGF, OS21+3_vcMMAE reduced cell viability to 22% (Fig. 5c), whereas 84% of cells remained viable after treatment with the ‘peptide-free’ DNA-vcMMAE complex (entry 7, Fig. S17). We conclude that the non-specific co-internalization of compounds promoted by HGF is inefficient.

Subsequent control experiments were conducted using Chinese Hamster Ovary (CHO) cells, which have been demonstrated to express the MET receptor but lack the EGFR.^56^ The peptide-DNA complexes OS21_vcMMAE and OS21+3_vcMMAE both offer the GE137 peptide for binding of MET. However, despite the presence of HGF as a factor for stimulating MET-dependent endocytosis, the bispecific DNA–peptide complexes showed low toxicity to the CHO cells (Fig. S18). For example, more than 95% of CHO cells (EGFR−, MET+) remained viable when treated with OS21_vcMMAE at 25 nM concentration, a value resembling the EC_50_ for cytotoxicity to A549 cells (EGFR+, MET+). Even at a 100 nM concentration of OS21_vcMMAE or OS21+3_vcMMAE, approximately 80% of the CHO cells remained viable. The low toxicity for CHO cells is indicative of the absence of a synergistic binding enhancement, which is a characteristic of the targeting of the MET/EGFR pair in A549 cells.

## Conclusions

The present study demonstrated the potential of DNA-programmed heterobivalent display of peptides and cyclopeptides for the enhanced targeting of cells expressing both the epidermal growth factor receptor (EGFR) and the mesenchymal-epithelial transition factor (MET). Peptides that had previously been used to target EGFR and MET separately were synthesized chemically and then conjugated with 20–21-nucleotide DNA strands *via* copper click reactions. As demonstrated by our results, this approach can be applied to relatively complex peptides containing two disulfide bridges, such as GE137, which is used for MET targeting. Nucleic acid hybridization provided a convenient means of assembling nicked duplexes, and afforded a collection of four monovalent and ten bispecific agents presenting both the 12-mer GE11 (recognising EGFR) and the bicyclic 26-mer GE137 peptide (recognising MET) at distances ranging from one to 41 base pairs, with and without the inclusion of unpaired nucleotides. A key finding of this study was the pronounced distance-affinity profile observed when staining live A549 cells, which express both EGFR and MET. Flow cytometry revealed that the highest affinity was obtained when GE11 and GE137 were presented from a complex formed by the hybridisation of 20- and 21-nucleotide (nt) long DNA–peptide conjugates with a 44-nt template strand. In this instance, the peptides were separated by 21 paired and three unpaired nucleotides. Assuming B-type duplex architecture and a rise of 1.2 Å per unpaired base,^29^ the distance can be estimated at approximately 73 Å. Substantial affinity losses were observed for complexes presenting the peptides at larger or smaller distances, with either more or fewer unpaired nucleotides, which illustrates the importance of linker optimization for the development of bispecific agents. Cell viability measurements performed with auristatin-armed DNA–peptide displays demonstrate that the optimized assemblies exhibit robust cytotoxicity in A549 cells in the presence of hepatocyte growth factor (HGF). The data reveal that both targeting and internalization are required for activity, and that conjugation with DNA effectively masks the cytotoxic payload until internalization occurs specifically.

In summary, our study demonstrates that self-assembly through nucleic acid hybridization is an effective strategy for optimizing bispecific agents capable of targeted cellular delivery. Notably, the DNA complexes described here are considerably smaller than previously reported bispecific agents based on DNA nanoarchitectures or antibody conjugates. Future avenues should consider using nuclease-stable oligonucleotide analogues for applications in animal experiments and heterobivalent presentation of peptide and cyclopeptide clusters to improve targeting through both chelate- and cluster-effect enhanced binding.

## Author contributions

PG: data curation, formal analysis, investigation, methodology, software, visualization, writing – original draft, HD: data curation, formal analysis, methodology, OS: conceptualization, funding acquisition, investigation, resources, supervision, validation, visualization, writing – review & editing.

## Conflicts of interest

There are no conflicts to declare.

## Supplementary Material

CB-OLF-D5CB00238A-s001

## Data Availability

The data supporting this article have been included as part of the supplementary information (SI). Supplementary information is available. See DOI: https://doi.org/10.1039/d5cb00238a.
