# Supplementary material for: DNA-programmed bispecific peptide assemblies for delivering cytotoxic payload to cells expressing EGFR and MET receptors
Source: RSC Chem Biol. 2025 Dec 3;7(2):200–7. doi: 10.1039/d5cb00238a (PMC12694606; doi:10.1039/d5cb00238a)
Supplement: CB-007-D5CB00238A-s001 [file CB-007-D5CB00238A-s001.pdf]

Supplementary Information (SI) for RSC Chemical Biology.
This journal is © The Royal Society of Chemistry 2025

Supporting Information

DNA-programmed bispecific peptide assemblies for delivering cytotoxic payload to cells expressing EGFR and MET receptors

Pritam Ghosh,[a] Huyen Dinh,[a] Oliver Seitz*[a]

[a] Dr. Pritam Ghosh, Dr. Huyen Dinh, Prof. Dr. Oliver Seitz
Institute of Chemistry
Humboldt-Universität zu Berlin
Brook-Taylor-Str. 2, D-12489 Berlin, Germany
E-mail: oliver.seitz@chemie.hu-berlin.de

A. Materials for Synthesis	2
B. Instrumentation	3
C. Automated solid phase peptide synthesis (SPPS)	4
D. Cyclization protocol for synthesis of bicyclic GE137	7
E. Synthesis of GE137-TAMRA	8
F. Conjugation of peptides with DNA	9
G. Conjugation of vcMMAE with DNA	9
H. Formation of dsDNA-peptide complexes	10
I. Cell Culture	13
J. Flow cytometry	14
K. Fluorescence Microscopy	19
L. Cell viability	22

A. Materials for Synthesis

Unless otherwise stated, all solvents and reagents were used as received from commercial suppliers. Fmoc-protected amino acid (AA) building blocks [Fmoc-Gly-OH, Fmoc-L-Ala-OH, Fmoc-L-Val-OH, Fmoc-L-Leu-OH, Fmoc-L-Ile-OH, Fmoc-D-Phe-OH, Fmoc-L-Phe-OH, Fmoc-L-His(Trt)-OH, Fmoc-L-Asn(Trt)-OH, Fmoc-L-Gln(Trt)-OH, Fmoc-L-Arg(Pbf)-OH, Fmoc-L-Lys(Boc)-OH, Fmoc-L-Lys(N_3)-OH, Fmoc-L-Tyr(tBu)-OH, Fmoc-L-Ser(tBu)-OH, Fmoc-L-Thr(tBu)-OH, Fmoc-L-Asp(OtBu)OH, Fmoc-L-Glu(OtBu)-OH, Fmoc-L-Cys(Trt)-OH, Fmoc-L-Cys(Acm)-OH, Fmoc-Pro-OH, Fmoc-L-Trp(Boc)-OH], O-(7-Azabenzotriazol-1-yl)-N,N,N′,N′-tetramethyluronium hexafluorophosphate (HATU), were purchased from Carbolution Chemicals (St. Ingbert, Germany). TentaGel® R RAM resin was obtained from Rapp Polymere GmbH (Tübingen, Germany) with 0.19 µmol/mg loading. Ethyl (2Z)-2-cyano-2-(hydroxyimino)acetate (Oxyma) and O-(1H-6-chlorobenzotriazole-1-yl)-1,1,3,3-tetramethyluronium hexafluorophosphate (HCTU) were purchased from Carbolution Chemicals GmbH (Saarbrücken, Germany). N,N-Diisopropylethylamine (DIPEA), N-methylpyrrolidone (NMP), 4-Methylmorpholine (NMM), Acetic anhydride (Ac_2O), and trifluoroacetic acid (TFA) were obtained from Carl Roth GmbH (Karlsruhe, Germany). 1H-1,2,3-Benzotriazol-1-ol (HOBt) was from abcr GmbH (Karlsruhe, Germany). N, N-Dimethylformamide (DMF) and acetonitrile (ACN) were purchased from VWR International GmbH (Darmstadt, Germany). Thionyl chloride was purchased from Sigma Aldrich. THPTA (Tris(3-hydroxypropyltriazolylmethyl)amine) ligand for copper click reaction was obtained from BLD Pharmatech GmbH (Reinbek, Germany). $CuSO_4 \cdot 5H_2O$ was purchased from Merck (Darmstadt, Germany). Ascorbic acid was purchased from Carl Roth. TAMRA alkyne, 5-isomer was purchased from Lumiprobe GmbH. Maleimide functionalized vcMMAE was purchased from BLD Pharmatech GmbH. Template strand gcgtaaggagatctggaatgagtcgagctccataataagcg with 5':Atto 565 and thiol, gcgtaaggagatctggaatgaatccagtcgagctccataataagcg (+5 spacer nucleotide) with 5':Atto 565, gcgtaaggagatctggaatgaatcgtcgagctccataataagcg (+3 spacer nucleotide)with 5':Atto 565, gcgtaaggagatctggaatgatgtcgagctccataataagcg (+1 spacer nucleotide) with 5':Atto 565, strand 1 5'-cgc tta tta tgg agc tcg ac-3' with alkyne modification on either position, strand 2 5'-tca ttc cag atc tcc tta cgc -3' with alkyne modification on either position are purchased from Biomers, Germany and used without further purification.

B. Instrumentation

Ultra performance liquid chromatography (UPLC) was performed by using an Acquity UPLC on an Acquity H-Class system (Waters, Milford, MA, USA) equipped with a PDA (λ = 210 nm) and QDa detector, using an Acquity UPLC CSH C18 (2.1 x 50 mm, 1.7 µm, 130 Å) column. The analyses were performed using the same solvent system A/B as in the semi-preparative HPLC, and the columns were thermostated to 50 °C. HRMS (High-resolution mass spectrometry) data was obtained by using a Waters Xevo G3 qTOF mass spectrometer (Milford, MA, USA) equipped with a Waters LC system. QTOF was calibrated with 5 mM sodium formate solution in propanol: water (90:10, v/v). Lock mass compound was leucine enkephalin with reference mass at m/z = 556.2766. The Waters LC system consisted of Acquity UPLC I-Class Binary Solvent Manager and I-Class Sample Manager. The instrument was operated using electrospray ionization in positive and negative mode. Agilent 1260 infinity II and infinity lab LC/MSD were also used for UPLC using CSH C18 (2.1 x 50 mm, 1.7 µm, 130 Å) column. Semi preparative HPLC for purification of oligonucleotide conjugates was carried out on a Gilson 1105 HPLC System (Gilson, Limburg, Germany), column: YMC-Triart C18 (12nm, S-µm, 150 x 10.0 mm ID). The mobile phase consisted of a binary mixture of A (0.1 M triethylammonium acetate buffer, pH = 7.4, aq.) and B (acetonitrile). HPLC analysis and purification of DNA-peptide conjugates was performed using Acquity UPLC Oligonucleotide BEH C18 Columns, (50 x 2.1 mm, 1.7 µm, 130 Å) at 50 °C and a binary mixture of A (0.1M triethylammonium acetate buffer, aq. pH: 7.4-7.5) and B (acetonitrile) was used. RP-HPLC purification of peptides was performed by using an Agilent Technologies 1100 Series HPLC system (Santa Clara, USA) with a Polaris C18 column (250 x 10 mm, 5 µm, pore size: 220 Å) from Varian at a flow rate of 6 ml/min with a binary mixture of mobile phase A (98.9 % H_2O, 1 % ACN, 0.1 % TFA) and B (98.9 % ACN, 1 % H_2O, 0.1 % TFA) in a linear gradient as described. For detection, a multiple wavelength detector operating at three wavelengths (λ_1 = 210 nm, λ_2 = 260 nm, and λ_3 = 280 nm) was used. To determine the peptide and DNA-peptide conjugate concentration, the optical density of the corresponding solutions was measured using a NanoDrop spectrophotometer (PeqLab, Erlangen, Germany) against the medium blank. MALDI-TOF mass spectra were measured on a Shimadzu Axima Confidence spectrometer (Shimadzu, Kyoto, Japan) in positive mode. Diammonium hydrogen citrate (AHC) in ultra-pure water (100 mg/mL) and 3-hydroxypicolinic acid (HPA) in ultra-pure water:ACN (1:1, v/v) with a concentration of 50 mg/mL were prepared. Then HPA:AHC=10:1, v/v was prepared and used further in matrix. Samples were measured in positive mode with an ion gate of 5000 Da and pulsed extraction adjusted to the respective

analyte mass. The laser was operated at 50 Hz and a power of 90-120. Between 200 and 600 single shots were accumulated per mass spectrum. The recorded spectra were calibrated with the single protonated ion signals of the oligonucleotide calibration standard (Bruker Daltonics, Bremen, Germany).

C. Automated solid phase peptide synthesis (SPPS)

Automated SPPS was performed using a MultiPep RS peptide synthesizer (Intavis, Cologne, Germany) fritted columns (10 mL) purchased from Carl Roth GmbH (Karlsruhe, Germany). Prior to use, the resin was allowed to swell for 10 minutes in DMF (800 µL).

Fmoc removal: treatment of resin with 20 % piperidine in DMF (800 µl) for 2x 5 min. Subsequently, the resin was washed with DMF (4x, 1000 µl).

Coupling: Fmoc-protected amino acids (8.25 eq.), dissolved in a solution of NMP containing OxymaPure (8 eq.), were transferred to an activation vessel. A solution of HCTU (8 eq.) and NMM (24 eq.) in NMP was added. The resulting solution (0.6. M in Fmoc-protected amino acid) was transferred to the resin. After 60 min the reactor was drained and the resin washed with DMF (3x, 1000 µl). The first amino acid was double-coupled with 45 min coupling time in each step.

Capping: treatment of resin with DMF:Ac_2O:lutidine (89:5:6 v/v/v, 800 µl) for 10 min. Subsequently, the resin was washed with DMF (3x, 1000 µl).

C.1 SPPS of GE11

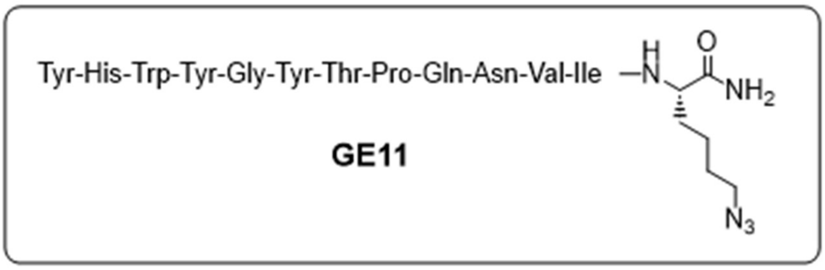

Fig. S1 GE11 amino acid sequence.

A fritted syringe reactor was charged with TentaGel® R RAM resin required for a 25 µmol scale. After Fmoc cleavage (20% piperidine in DMF, 800 µl, 2x 5 min) and washing with DMF (3x, 1 mL), the resin was treated with a mixture of 4 eq. of Fmoc-Lys (N_3)-OH, 3.85 eq. HATU and 8 eq. DIPEA in DMF (concentration: 0.2M). Subsequently, the resin was washed thoroughly with DMF (5x) and attached to the synthesizer for further couplings via automated SPPS (see C). After completion of peptide assembly, the Fmoc cleavage (20% piperidine in DMF, 800 µl, 2x

5 min) was performed followed by washing with DMF (3x, 1 mL), DCM (5x, 1 mL). The resin was dried under a vacuum. The global deprotection was performed with the 2 mL cleavage cocktail TFA/triisopropylsilane/H_2O 95:2.5:2.5 (v/v/v) at room temp for 2 h. After 2 h the solution was collected by filtration into 15 mL falcon tube. The resin was washed with the cleavage cocktail (3x 1 mL) and the combined filtrates were concentrated (~ 0.5 mL) under airflow. Diethyl ether (Et_2O) (10-fold volume) was added to the remaining residue. The suspension was cooled (in dry ice for ~30 min) and centrifuged (4000 rpm, 15 min). Afterwards, the ether phase was decanted. The remaining peptide pellet was dissolved in 1 mL of ACN: water (1:1, v/v with 0.1% TFA) by vortexing. The solution in the falcon tube was frozen by liquid nitrogen in a Dewar flask and was lyophilized. Further purification was performed by RP-HPLC. Preparative HPLC 30-90% B in 11 min with 6 mL/min flow rate. $C_{81}H_{108}N_{22}O_{19}$ (MW = 1693.89 g/mol). Yield: 18 mg (10.63 µmol), 53%. ESI-MS (pos. mode): m/z = 848.06 $(M+2H)^{2+}$, calcd.: 847.95 (Fig. S2).

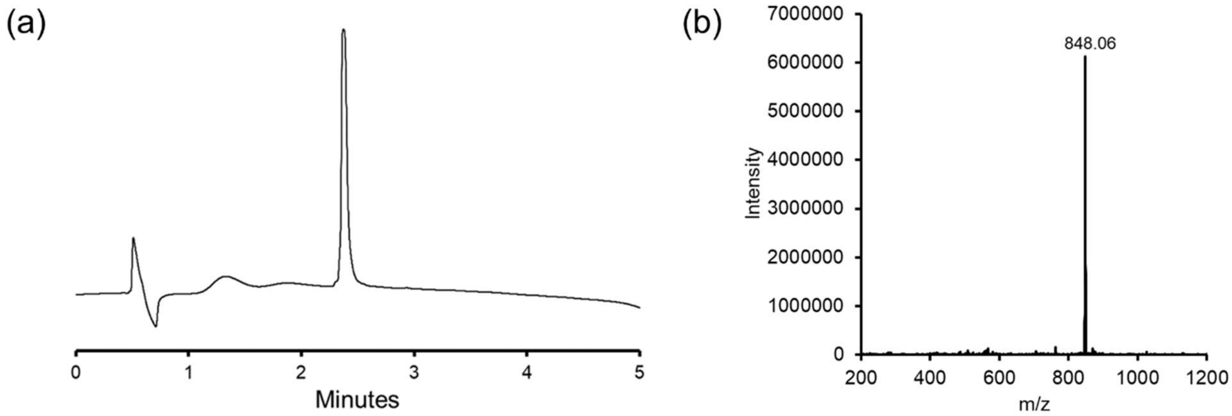

Fig. S2 (a) UPLC trace of purified GE11 at λ = 210 nm [3-80% solvent B in 6 min, column UPLC CSH C18 (2.1 x 50 mm, 1.7 µm, 130 Å]; (b) ESI-MS of purified GE11.

C.2 SPPS of linear GE137 precursor

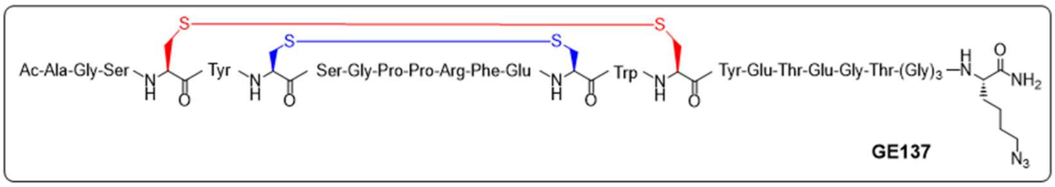

Fig. S3 GE137 amino acid sequence.

A fritted syringe reactor was charged with TentaGel® R RAM resin required for 25 µmol scale. After Fmoc cleavage (20% piperidine in DMF, 800 µl, 2x 5 min) and washing with DMF (3x, 1

mL), the resin was treated with a mixture of 4 eq. of Fmoc-Lys (N_3)-OH, 3.85 eq. HATU and 8 eq. DIPEA in DMF (concentration: 0.2 M). Subsequently, the resin was washed thoroughly with DMF (5x) and attached to the synthesizer for further couplings via automated SPPS (see C). After completion of the chain assembly, Fmoc cleavage (20% piperidine in DMF, 800 µl, 2x 5 min) was performed followed by washing with DMF (3x, 1 mL). The *N*-terminus was acetylated using acetic anhydride: lutidine: DMF (4:4:2, v/v/v, 1 mL) for 20 minutes two times. Subsequently, the resin was washed with DMF (3x, 1 mL), DCM (5x, 1 mL) and dried under vacuum. The global deprotection was performed with the 2 mL cleavage cocktail TFA/triisopropylsilane/H_2O 95:2.5:2.5 (v/v/v) at room temp for 2h. After 2 h the solution was collected by filtration into 15 mL falcon tube. The resin was washed with the cleavage cocktail (3x 1 mL) and the combined filtrates were concentrated (~ 0.5 mL) under airflow. Et_2O (10-fold volume) was added to the remaining residue, the suspension was cooled (in dry ice for ~30 min) and centrifuged (4000 rpm, 15 min). Afterwards, the ether phase was decanted. The remaining peptide pellet was dissolved in 1 mL of ACN: water (1:1, v/v with 0.1% TFA) by vortexing. The solution in the falcon was frozen by liquid nitrogen in a Dewar flask and was lyophilized. Further purification was performed by RP-HPLC.

Preparative HPLC 5–80% B in 40 min, peak appeared at ~18 min. $C_{124}H_{176}N_{36}O_{41}S_4$ (MW = 2955.22 g/mol). Yield: 14 mg (4.74 µmol), 40%. ESI-MS (pos. mode): m/z = 985.86 $(M+3H)^{3+}$, calcd.: 986.08 (Fig. S4).

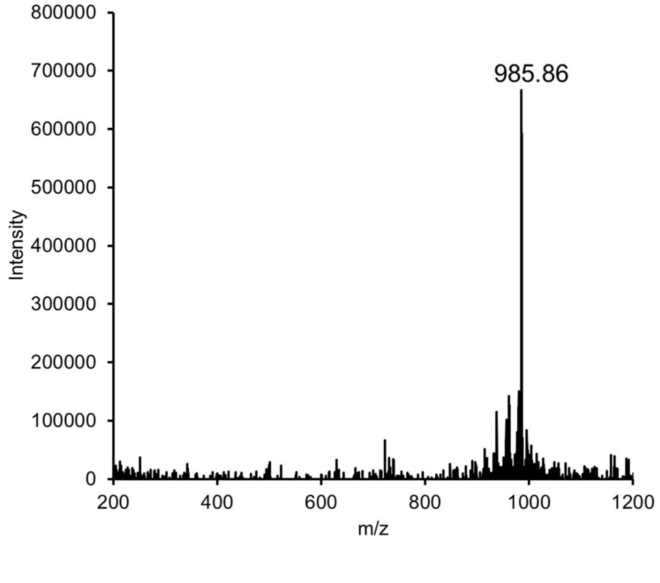

Fig. S4 UPLC trace of purified linear GE137 precursor at λ = 210 nm [3-80% solvent B in 6 min, column UPLC CSH C18 (2.1 x 50 mm, 1.7 µm, 130 Å] is provided in Fig. 2c (main text); the ESI-MS is shown here.

D. Cyclization protocol for synthesis of bicyclic GE137

Stock solutions used for synthesizing GE137:

(a) $PdCl_2$ (23 mg) in degassed 6 M Gn·HCl buffer (200 µL, 648.5 mM), pH 7;

(b) Sodium dithiocarbamate (22.5 mg) in water (100 µL, 1 M);

(c) disulfiram (30 mg) in ACN (300 µL, 336.7 mM);

(d) glutathione (15.36 mg) in water (100 µL, 0.5 M).

The lyophilized peptide (10 mg) o was dissolved in 6 M Gn·HCl buffer pH 7 (400 µL, 8.45 mM), and treated with DSF (75 µL from stock (c) for 5 minutes at 42°C. The mixture was submitted to RP-HPLC purification. Compound containing fractions were collected and lyophilized.

A portion (3 mg) was withdrawn from the lyophilizate and dissolved again solubilized in 6 M Gn.HCl buffer pH 7 (400 µL, 2.54 mM), followed by addition of 10 eq. $PdCl_2$ (~ 16 µL from stock (a)). The reaction mixture was kept at shaking (160 RPM) for 15 min at 42°C. Subsequently, 50 eq. DTC (~51 µL from stock (b)) and 10 eq. GSH (~20 µL from stock (d)) were added. After 15 min at 42°C at shaking (160 RPM), 10 eq. DSF (30 µL from stock (c)) was added. The reaction mixture was, again, kept for 15 min at 42°C at shaking (160 RPM). Subsequently, the peptide was purified using HPLC and lyophilized.

Preparative HPLC 5-70% B in 12.5 min. $C_{118}H_{162}N_{34}O_{39}S_4$ (MW = 2809.03 g/mol). Yield (for step Ox-2): 4 mg (1.42 µmol), 24%. ESI-HRMS (pos. mode): m/z = 1405.5402 $(M+2H)^{2+}$, calcd.: 1405.525, 937.3637 $(M+3H)^{3+}$, calcd.: 937.3533 (Fig. S5).

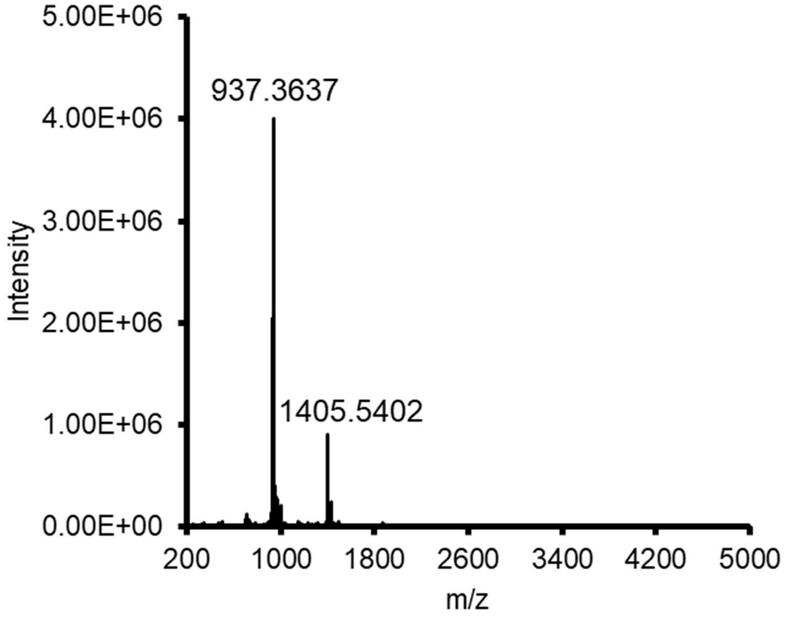

Fig. S5 HRMS data of GE137 purified peptide, calculated mass with two S-S bonds $C_{118}H_{162}N_{34}O_{39}S_4$ (MW = 2809.03 g/mol), ESI-HRMS (pos. mode): m/z = 1405.5402 $(M+2H)^{2+}$,

calcd.: 1405.525, 937.3637 $(M+3H)^{3+}$, calcd.: 937.3533. The UPLC trace of purified GE137 is shown in the main text Fig. 2i.

E. Synthesis of GE137-TAMRA

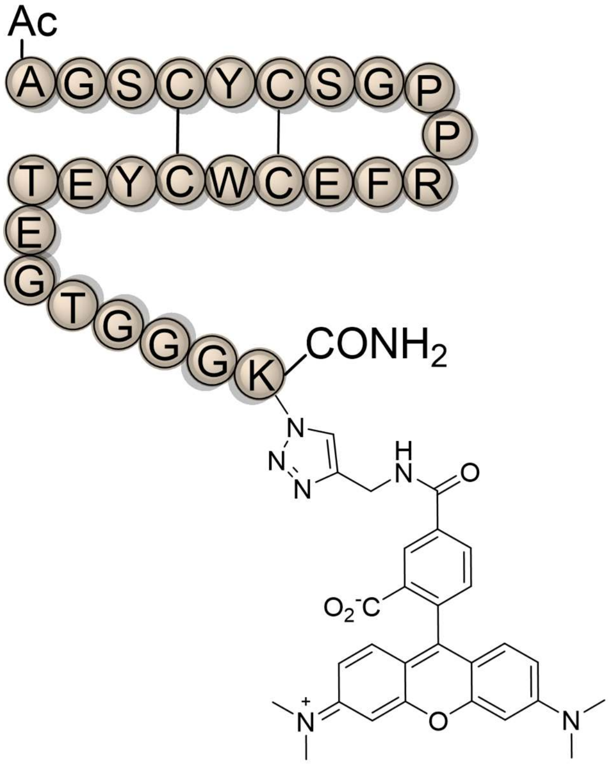

Fig. S6 Chemical structure of TAMRA linked to GE137 by copper click reaction (UPLC and mass trace are shown in Fig. S7). The synthesis of GE137-TAMRA was previously described in Ref 1.

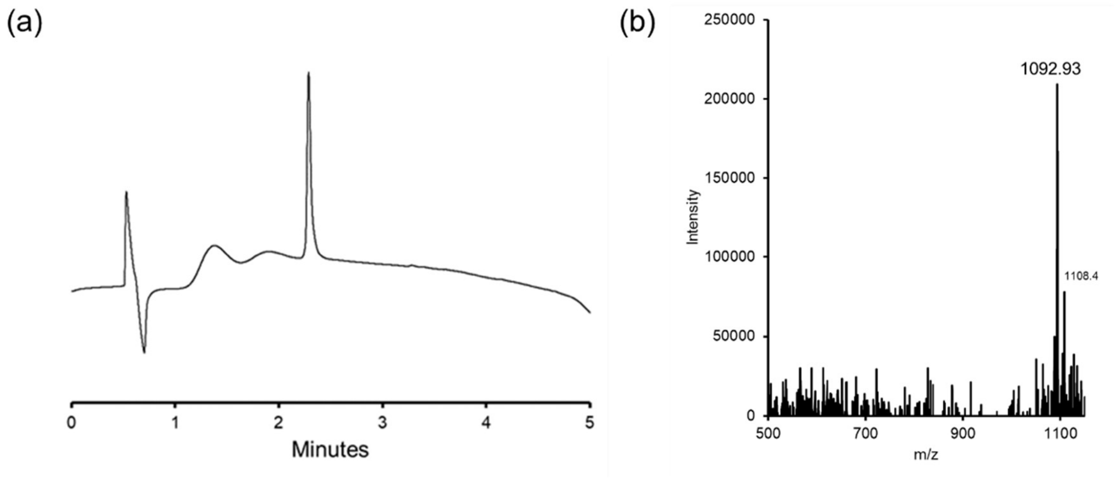

Fig. S7 (a) UPLC analysis of GE137-TAMRA at λ = 210 nm [3-80% solvent B in 6 min, UPLC CSH C18 (2.1 x 50 mm, 1.7 µm, 130 Å]; (b) $C_{146}H_{187}N_{37}O_{43}S_4$ (MW = 3276.53 g/mol), ESI-MS analysis: m/z = 1092.93 $(M+3H)^{3+}$, calcd.: 1093.18.

F. Conjugation of peptides with DNA

The conjugation and characterization data of obtained conjugates was previously described in Ref 1. The following conjugates were used:

5’-**GE11**-C*GCTTATTATGGAGCTCGAC-3’
5’-**GE137**-T*CATTCCAGATCTCCTTACGC-3’
5’-CGCTTATTATGGAGCTCGAC*-**GE11**-3’
5’-TCATTCCAGATCTCCTTACGC*-**GE137**-3’

Conjugation sites are marked with asterisk. The precise structure of terminal modifications is:

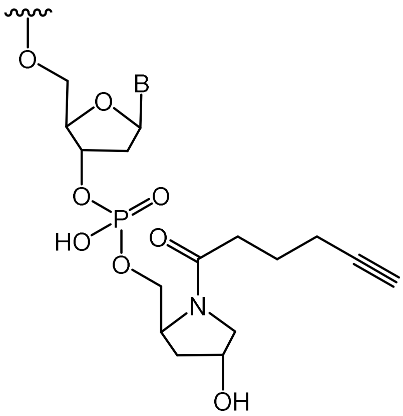

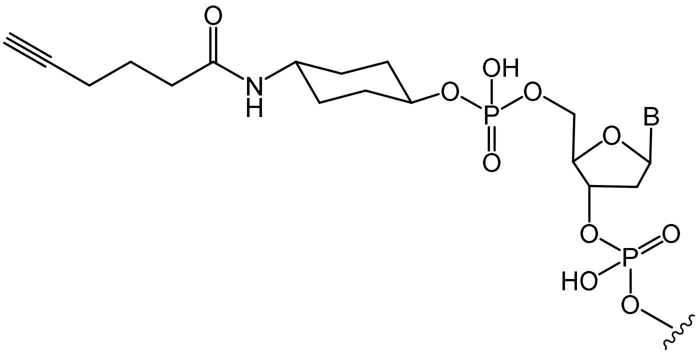

3’-end modification 5’-end modification

G. Conjugation of vcMMAE with DNA

Conjugation was performed in reactions of maleimide modified vcMMAE with thiolated DNA template strands.

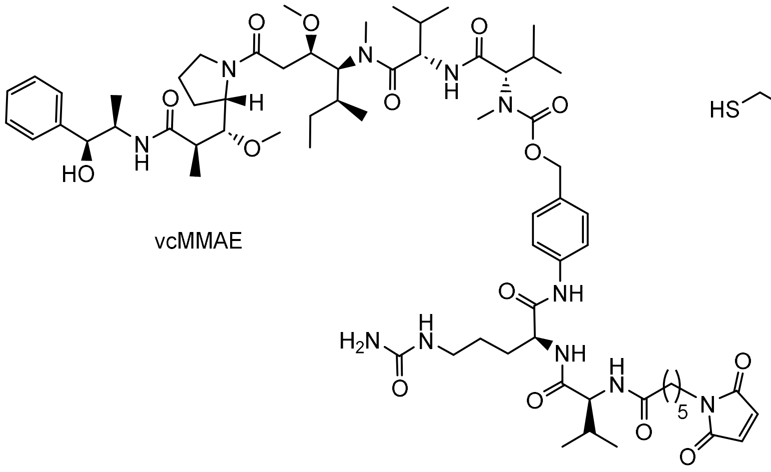

vcMMAE

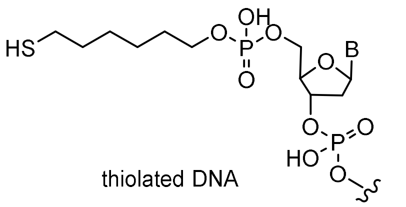

thiolated DNA

The following conjugates were used:

5´-**vcMMAE**-G*CGTAAGGAGATCTGGAATGAGTCGAGCTCCATAATAAGCG-3´
The synthesis and characterization data of this conjugate was previously described in Ref 1.

5´-v**cMMAE**-G*CGTAAGGAGATCTGGAATGAATCGTCGAGCTCCATAATAAGCG-3´

0.1 M phosphate buffer (pH 7.4) was degassed by purging argon and freezing in liquid nitrogen. The thiol-modified template strand was incubated with 100 mM tris(2-carboxyethyl) phosphine (TCEP) for 30 min at 42°C in 0.1 M phosphate buffer (pH 7.4) to reduce possible disulfide bonds. Subsequently, the maleimide functionalized vcMMAE dissolved in DMSO (0.5 M) was added in 10-fold molar excess. The final concentration of the oligonucleotide in the reaction mixture was 200 µM. The reaction was allowed to proceed overnight at 37 °C. The product was purified by HPLC, freeze-dried, and stored at −20 °C until use. Prior to use, the conjugate was dissolved in water, and the final concentration was determined photometrically at 260 nm assuming extinction coefficients calculated by https://molbiotools.com/dnacalculator.php.

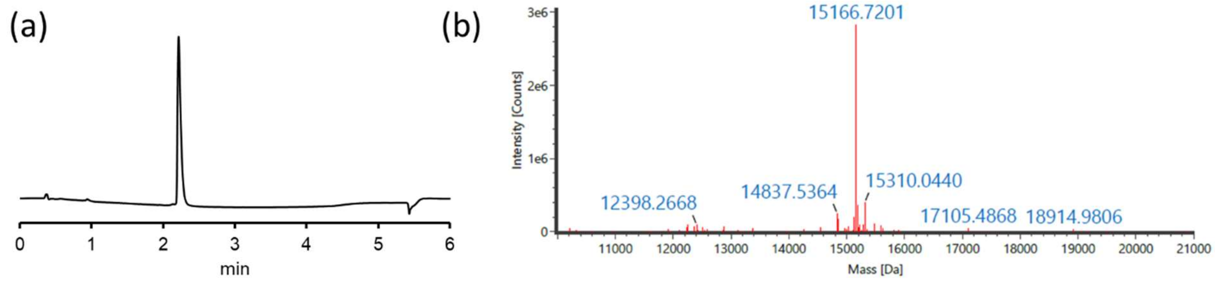

Fig. S8 (a) UPLC trace at λ = 260 nm (t_R: 2.21 min, 3-80% solvent B in 6 min) of the 46 nt long template-vcMMAE conjugate. (b) MALDI-TOF-MS analysis (performed at the facility of Biomers GmbH, Germany); Calculated mass: 15166.63 Da, found: 15166.72 Da in MALDI.

H. Formation of dsDNA-peptide complexes

Template strand and shorter strands were mixed in equimolar stoichiometry with a ratio of 1:1:1, resulting in a final concentration of 10 µM in Milli-Q® water. After 2 min at 90 °C, the solution was rapidly cooled down to room temperature. This stock solution was diluted with buffer used for the cell experiments.

H. 1 Native Polyacrylamide Gel Electrophoresis (native PAGE)

Bispecific DNA complexes were characterized through native polyacrylamide gel electrophoresis (native PAGE) at a concentration of 20% acrylamide/bisacrylamide (19:1 v/v). All samples were loaded at a concentration of 50 ng/µl upon mixing with 6x purple gel loading dye (New England Biolabs). The electrophoresis was performed in 1x TBE buffer at a constant voltage of 90 V for 1h at room temperature. The gel was stained with SYBR Gold (1:10,000) in 1x TBE buffer for 15 min while gently rocking at room temperature and were imaged again (Gel Doc XR+, Bio-Rad), see Fig. S9.

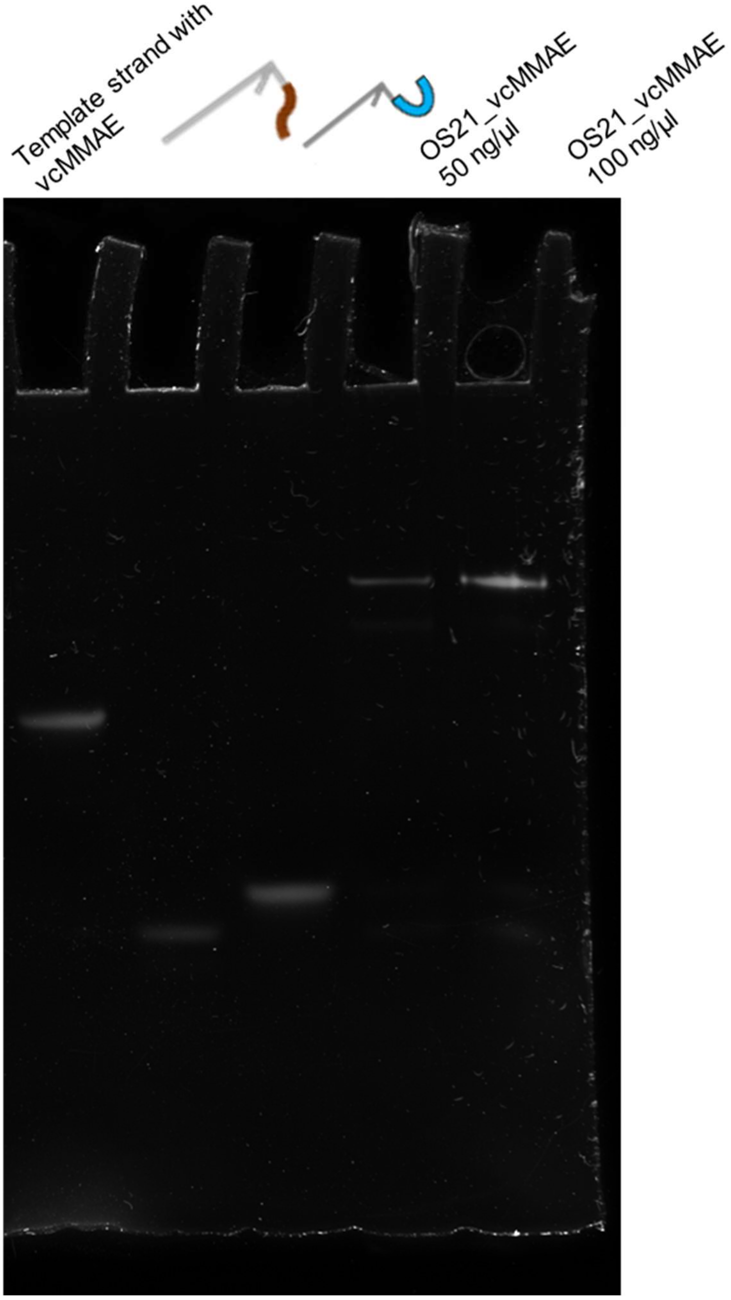

Fig. S9 Native PAGE for characterizing duplex formation of the bispecific DNA probes visualized by SYBR gold staining.

Table S1. Structure and abbreviation of the dsDNA complexes used in the work.

SI No.	DNA constructs	Structural Abbreviation
1<br>5'S-41	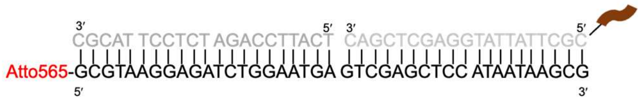	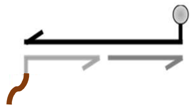
2<br>3'S-41	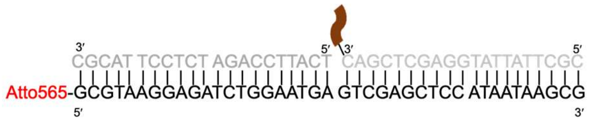	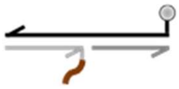
3<br>3'O-41	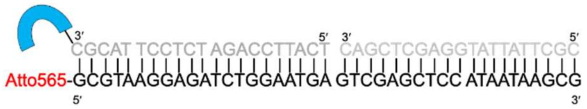	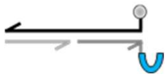
4<br>5'O-41	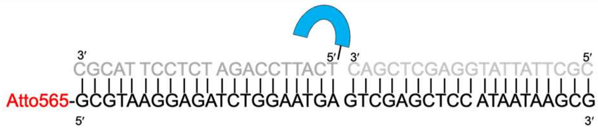	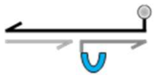
5<br>OS-1	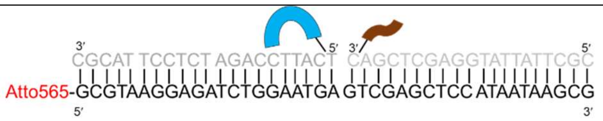	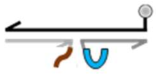
6<br>OS-20	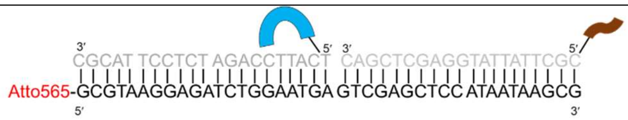	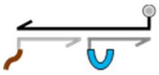
7<br>OS-21	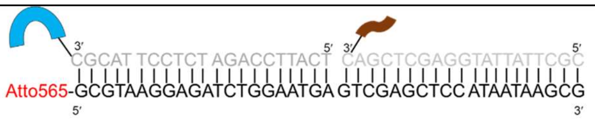	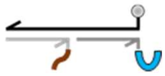
8<br>OS-40	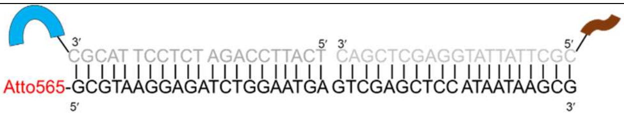	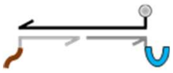
9<br>OS-20+1	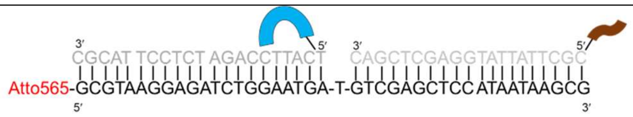	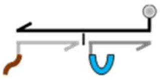
10<br>OS-20+3	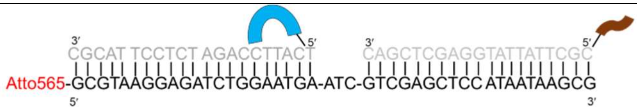	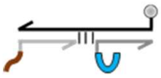
11<br>OS-20+5	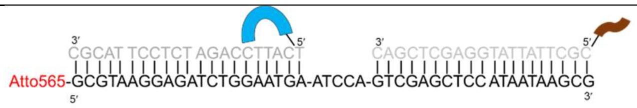	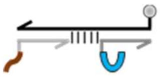

12<br>OS-21+1	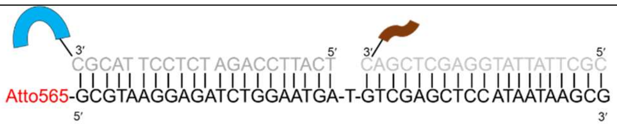	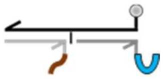
13<br>Os-21+3	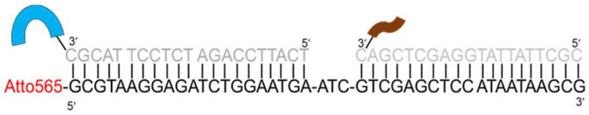	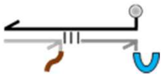
14<br>OS-21+5	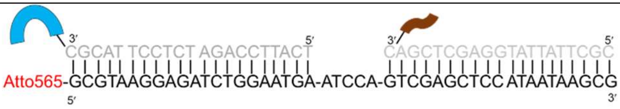	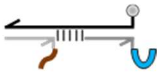
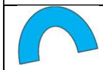 : GE137 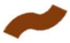 : GE11		

I. Cell Culture

I.1 Reagents, media for cell culture

Dulbecco’s Modified Eagle medium (DMEM) High Glucose, Ham’s F12 medium and RPMI 1640 medium were purchased from Biowest (Nuaillé, France). Fetal Bovine Serum (FBS), SYTOX™ Blue Dead Cell Stain, alamarBlue™ Cell Viability Reagent, StemPro™ Accutase™ Cell Dissociation Reagent, Poly-D-Lysine (0.1 mg/ml) and Hoechst 33342 (10 mg/ml), were obtained from Thermo Fisher Scientific (Waltham, USA). Accutase® enzyme in Dulbecco's PBS containing 0.5 mM EDTA (without calcium and magnesium) was purchased from Biolegend (California, USA). Sterile 10x PBS pH 7.4 without Ca/Mg was purchased from Carl Roth GmbH (Karlsruhe, Germany). A549 cells were obtained from ATCC (Virginia, USA) and pre-screened single donor C-12205 Promocell. Trypsin-EDTA 1X in PBS without Ca/Mg without Phenol Red was purchased from VWR International GmbH (Darmstadt, Germany). Penicillin/Streptomycin (10,000 U/mL) was obtained from MP Biomedicals Germany GmbH (Eschwege, Germany). µ-Slide 8 Well microscopy slides were purchased from ibidi GmbH (Gräfelfing, Germany). Perkin Elmer Victor plate readers were used in fluorescence channel for alamar blue™ as suggested by the vendor’s protocol. HEPES, NaCl and $CaCl_2$ were obtained from Sigma (St. Louis, Missouri, USA). Lysotracker Blue was obtained from ThermoFischer (Waltham, USA) and used as suggested by the vendor’s protocol. HGF was purchased from Peprotech (Cranbury, New Jersey, USA).

I.2 Preparing the medium

For culturing A549 cells, DMEM/Ham's F12 1:1 (v/v) with 10% FBS and 1% penicillin/streptomycin was prepared. The medium was always preheated to 37°C before use.

I.3 Thawing cells

Before thawing the cells, a T75 culture flask was charged with 10 mL medium and warmed to 37°C in the incubator. The cryovial was removed from the nitrogen tank and allowed to reach room temperature, then transferred to a prewarmed water bath at 37°C for thawing. The cell suspension was transferred to a centrifuge tube containing pre-warmed medium and spin down at 200xg for 5 minutes to wash off residual freezing medium containing DMSO. Then, the resuspended cells were transferred to the prepared T75 culture flask and incubated at 37°C and 5% CO_2. At a confluence of 80-90%, the cells were passaged.

I.4 Culture of A549 cells

A549 cells were cultured in DMEM/Hams F12 1:1 (v/v) with 10% FBS and 1% penicillin/streptomycin at 37°C and 5% CO_2. Once confluency reached between 85-95%, the supernatant was aspirated, and cells were washed with PBS pH 7.4 to remove the residual medium. For cell detachment, 1 mL accutase was added, and the cells were incubated at 37°C with 5% CO_2 for five minutes. To stop the accutase activity, fresh medium was added, and the A549 cells were transferred to a 15 ml tube and centrifuged. Passages 6-9 were preferably used during analysis (ref 2). After counting the cells, one part of the suspension was seeded for further culturing, while another portion was used for experiments.

J. Flow cytometry

As explained in sections I.4, after counting the cells, one part of the suspension in respective full medium was seeded for further culturing, while another portion was used for experiments. This second portion (while the cells are in suspension in respective culture medium) was used in flow cytometry. Here this cell suspension was centrifuged 200xg for 5 min and the cell pellet was resuspended in DNA-peptide conjugate for incubation. Flow cytometry experiments were performed on a BD Accuri™ C6 (BD Biosciences). Excitation lasers: YFP, 488 nm; Atto647N, 640 nm. Emission filters: YFP, 533 ± 15 nm; Atto647N, 675 ± 12.5 nm. 10000 cells were counted in each case and the median value was taken from the instrument, which was used further

for plotting data. The data was further processed using FlowJo™ software. The gating strategy is provided for all the cell lines in Fig. S10.

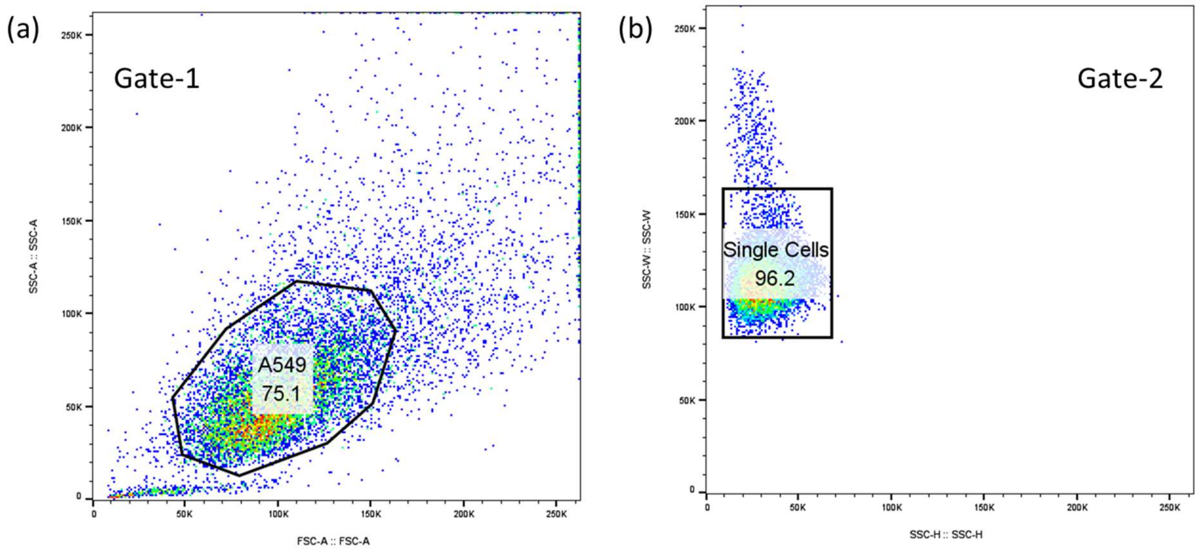

Fig. S10 Exemplified gating strategy for A549 cells applied in flow cytometry analysis excluding cells debris (FSC-A, SSC-A; Gate 1) and doublets (SSC-H, SSC-W; Gate 2). Approx. 10,000 cells per sample were analysed. Channels used are: Atto-565 fluorescence intensity for experiments with DNA-peptides or only peptide-TAMRA probes.

J1. Staining with GE137-TAMRA:

The peptide-TAMRA conjugate (200 nM in PBS) was added to A549 cells. After 10 minutes of incubation at 37°C the cells were centrifuged and measured in PBS. In a second experiments, dye-free GE137 (500 nM in PBS) was added to the cells and incubated for 10 minutes at 37°C. After centrifugation at (200G at 37°C) , the medium was removed and replaced with a solution of GE137-TAMRA in PBS (200 nM). The cells were incubated for 10 minutes at 37°C, centrifuged and measured in PBS. As a control, cells were also incubated in PBS for 20 minutes and measured (Fig. S11).

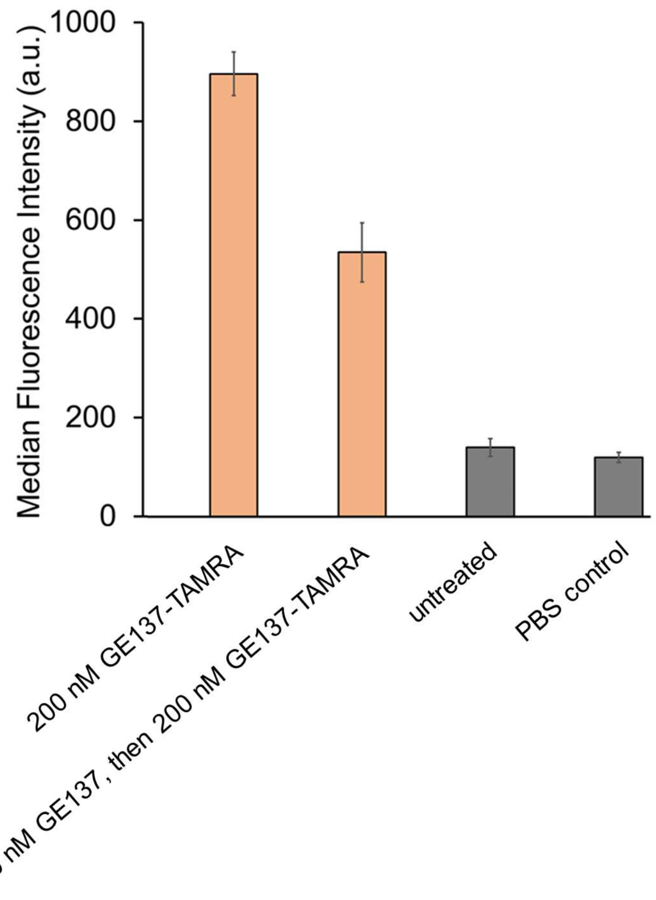

Fig. S11. Binding of GE137-TAMRA to A549 cells assessed by flow cytometry. Incubation time: 10 minutes in PBS, temp: 37 °C, 5 % CO_2, flow cytometry was performed with suspended cells, 10000 cells were counted in each case and the median value was plotted.

J2. Staining with DNA-Peptide conjugates

Solutions of DNA-peptide conjugates are prepared at 10 µM concentration as stock in MilliQ water and subsequently diluted in PBS. Cells are taken as suspension in respective culture medium which was centrifuged 200xg for 5min and the cell pellet was resuspended with DNA-peptide conjugate solution in PBS, incubated at 37°C, 5%CO_2 for 10 minutes. After 10 minutes, the cells are centrifuged 200xg for 5min, washed carefully with PBS once by resuspension. Further centrifuged 200xg for 5min and resuspended in PBS for the experiments.

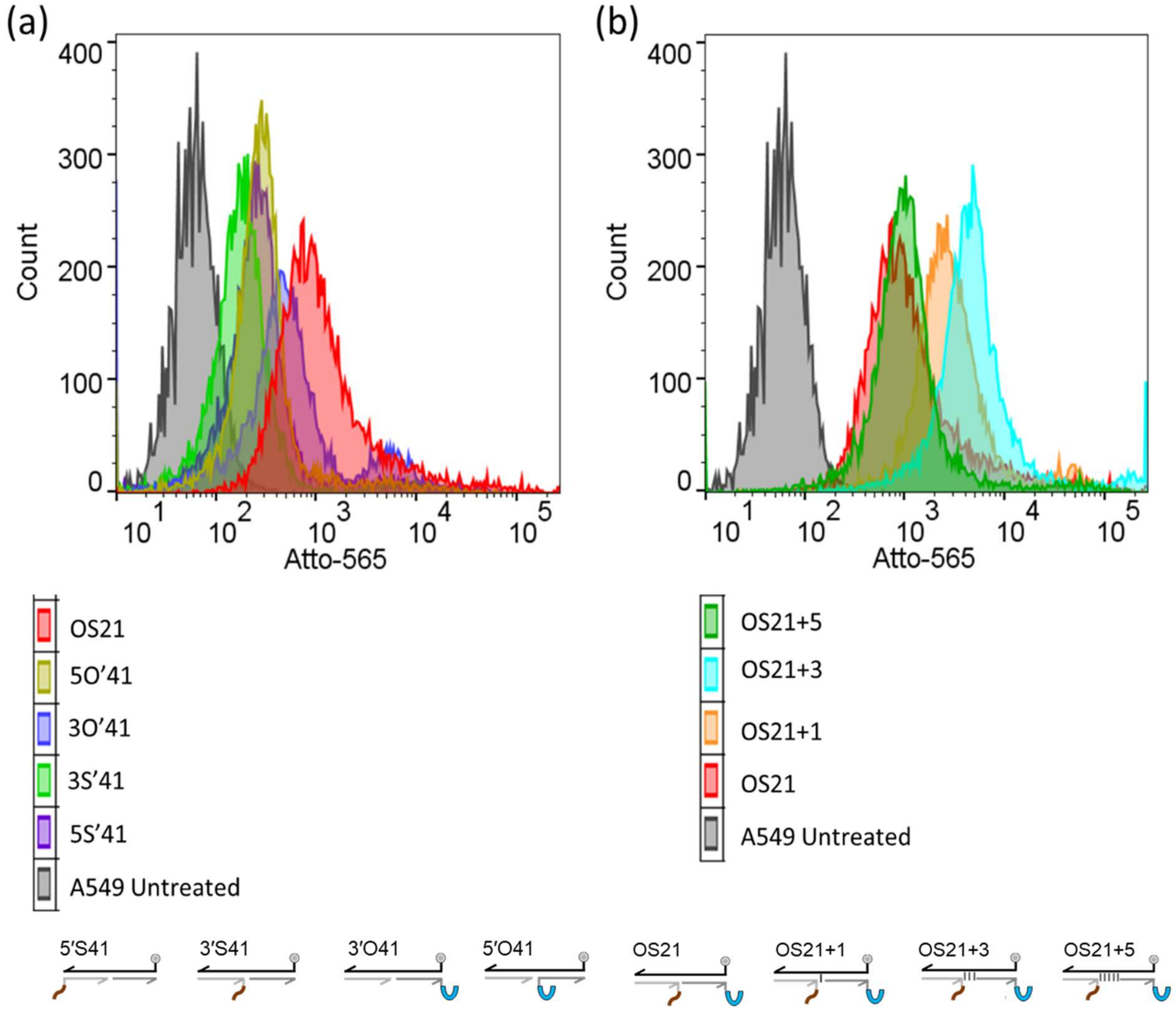

Fig. S12. Staining of A549 cells with monovalent and bispecific DNA-peptide complexes assessed by flow cytometry of live cells. Conditions: 750 nM, 10 min incubation in PBS at 37 °C, 5 % CO_2, flow cytometry was performed with suspended cells, 10000 cells were counted in each case and the median value was plotted.

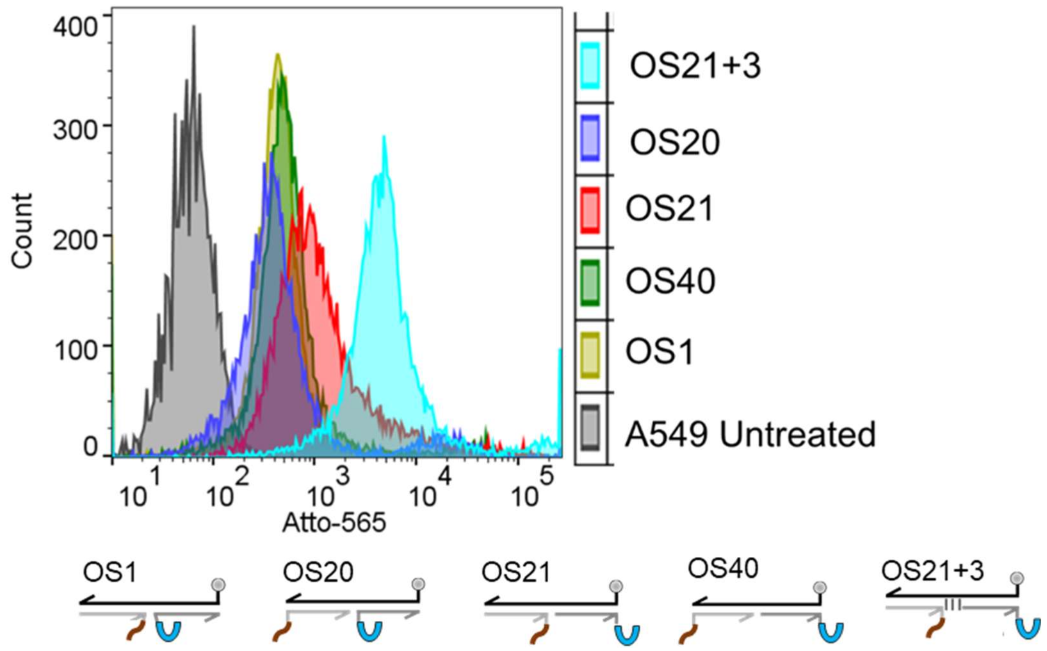

Fig. S13. Staining of A549 cells with bispecific DNA-peptide complexes assessed by flow cytometry of live cells. Conditions: 750 nM, 10 min incubation in PBS at 37 °C, 5 % CO_2, flow cytometry was performed with suspended cells, 10000 cells were counted in each case and the median value was plotted.

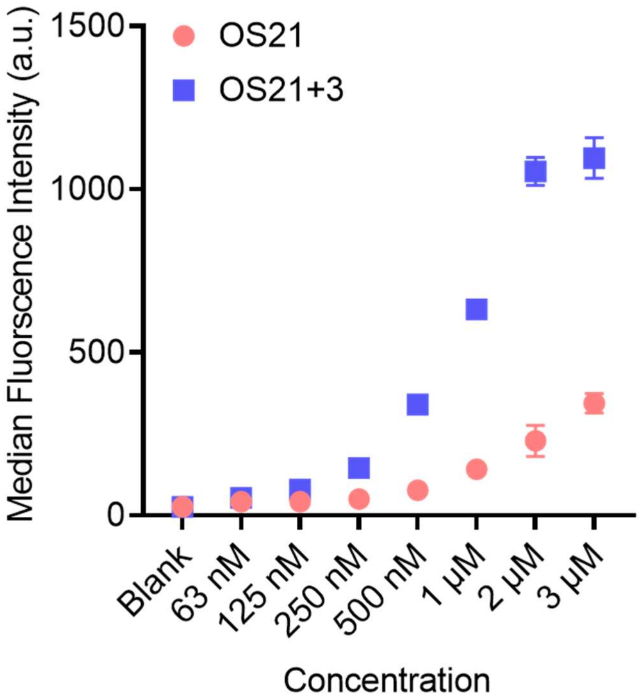

Fig. S14. Concentration dependent binding of OS21 and OS21+3 peptide-DNA complexes to A549 cells, assessed by flow cytometry. Conditions: 10 min incubation, 37 °C, 5 % CO_2, PBS.

K. Fluorescence Microscopy

Wide-field fluorescence microscopy experiments were performed using an IX83 microscope from Olympus with a 60x magnification lens. The channels used were: Blue (Hoechst33342): λ_{ex} = 350 ± 25 nm, λ_{em} = 460 ± 25 nm; Red (Atto-565): λ_{ex} = 575 ± 12 nm, λ_{em} 628 ± 20 nm. Cell Sens Dimension V1.17 (Olympus) software was used for image analysis. A549 cells (50.000 per well) were seeded and cultured for 48 hours in [DMEM/Hams F12 1:1 (v/v) with 10% FBS and 1% penicillin/streptomycin] on µ-slides (ibidi). The medium was then removed, and the cells were washed twice with PBS preheated to 37°C. Probes [OS21 without HGF for Fig. S15, OS21+3 with HGF for Fig. 4a-e (zoomed in version at 5µm scale) and Fig. S16a-d (at 20µm scale) and OS21 with HGF for Fig. 4f-j (zoomed in version at 5µm scale) and Fig. S16e-h (at 20µm scale) (1 µM, 200 µL)] were added and incubated for 30 minutes at 37°C with 5% CO_2. After incubation, the cells were washed three times with PBS. Nucleus staining was performed using Hoechst 33342 (2 µg/mL, 200 µL) and Lysotracker (100nM in PBS) at 37°C with 5% CO_2 for 10 minutes. Finally, the cells were washed once with PBS and prepared for microscopy (Fig. S15 and S16).

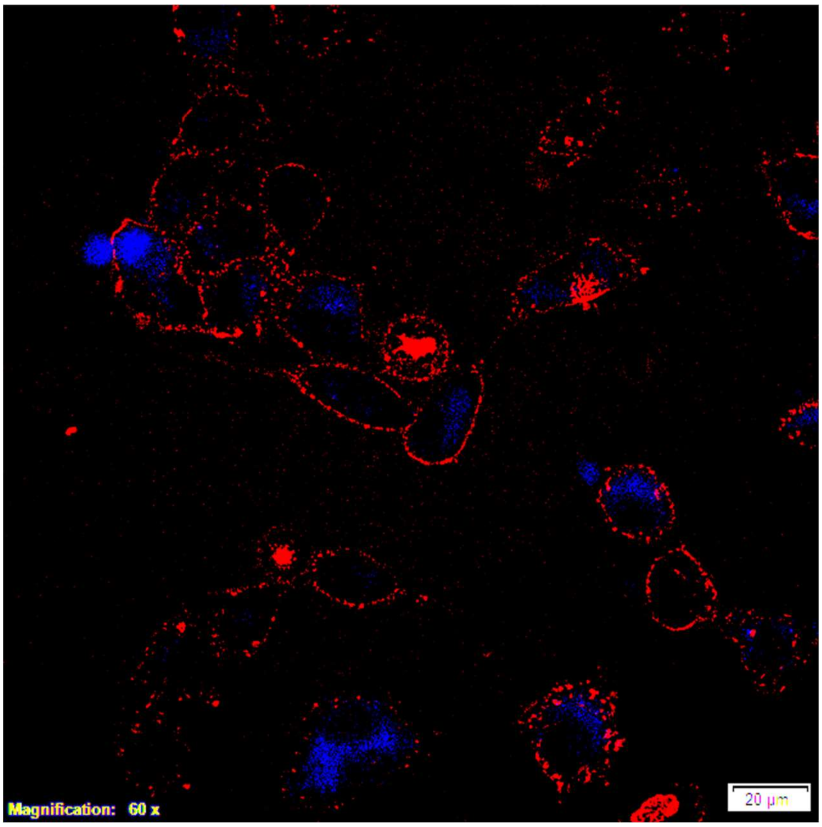

Fig. S15 Microscopy images of A549 cells after staining with OS21. Conditions: 1 µM OS21 in PBS, 30 minutes incubation time, 37 °C, 5 % CO_2. scale bar: 20 µm; medium: PBS, 50.000 cells seeded per well. Blue: nuclear labelling with Hoechst33342 (λex = 350 ± 50 nm, λem = 460 ± 50 nm); Red: Signal of Atto-565 in TRITC channel (λex = 575 ± 25 nm, λem > 593 nm). The image was deconvoluted using the cellSens dimension software and the 2D deconvolution function.

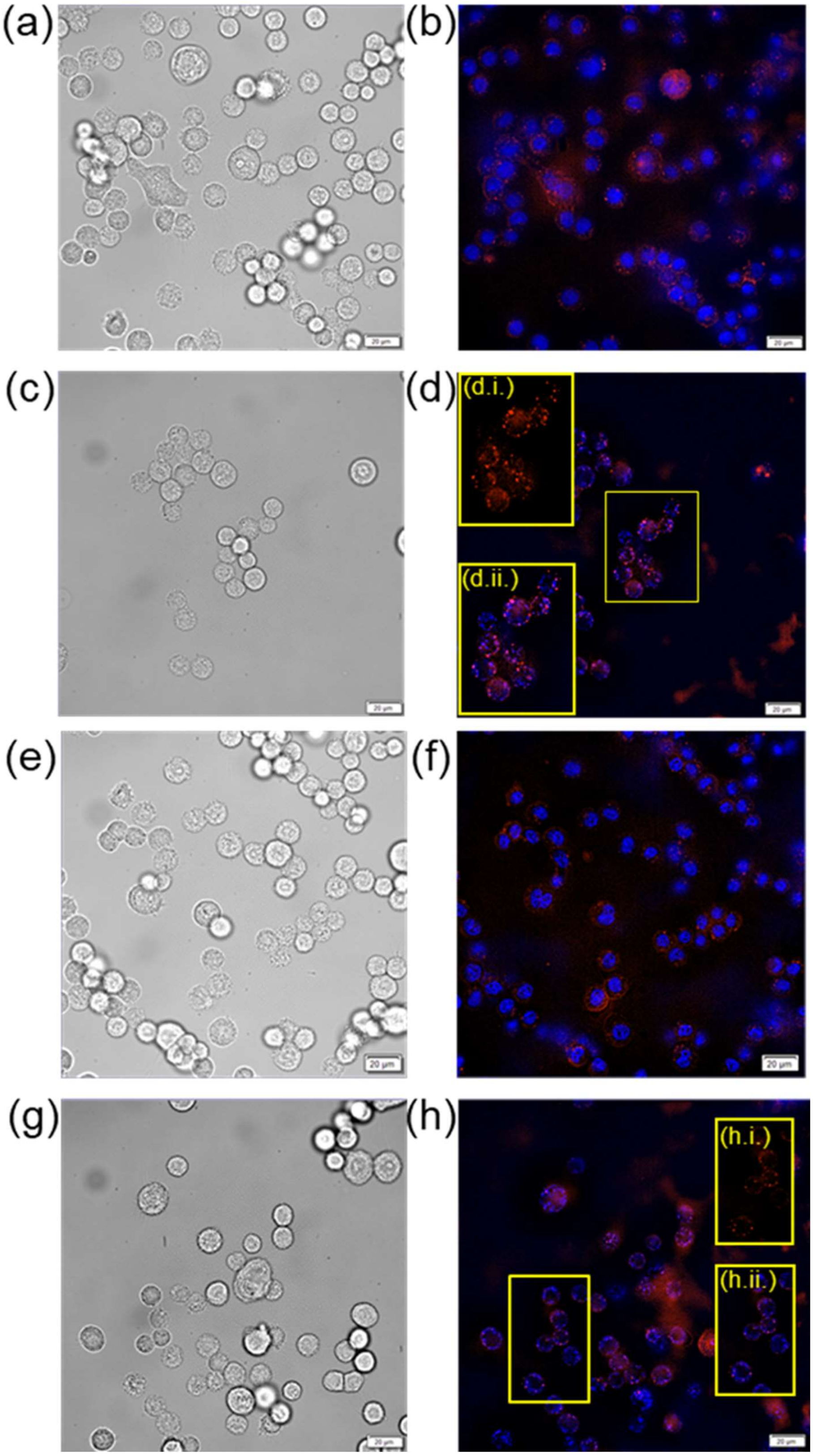

Fig. S16 Microscopy images of A549 cells after staining with (a-d) OS-21+3 and (e-h) OS-21: Co-staining was performed for nuclei with Hoechst33342 (b: OS-21+3 and f: OS-21), or lysomes with lysotracker (d: OS-21+3 and h: OS-21). The rectangular boxes in (d) and (h) highlight

magnified areas analyzed by co-staining with lysotracker (d.i. and h.i. show red fluorescence emitted by Atto565-labelled DNA-peptide complexes, d.ii. and h.ii. show overlay with blue fluorescence from lysotracker, where purple suggests overlap). Conditions: 1 µM probe in PBS, 30 minutes incubation time, 37 °C, 5 % CO_2. scale bar: 20 µm; medium: PBS, 50.000 cells seeded per well. Blue: nuclear labelling with Hoechst33342 or lysotracker (100nM in PBS) ($\lambda_{ex} = 350 \pm 50$ nm, $\lambda_{em} = 460 \pm 50$ nm); Red: Signal of Atto-565 in TRITC channel ($\lambda_{ex} = 575 \pm 25$ nm, $\lambda_{em} > 593$ nm).

L. Cell viability

A549 cells were seeded into wells of a 96 well-microtiter plate (100 µL in respective medium) at a density of 10^4 cells/well. Cells were allowed to grow for 24 h to a confluency of ca. 85%. The medium was removed and wells carefully washed once with PBS (preheated at 37°C). The probes were added at specific concentrations in PBS with or without HGF (100 ng/mL). The cells were incubated at 37°C, 5% CO_2 for 10 minutes. After one careful wash with PBS, 10% alamar blue was added, and the fluorescence signal (590 nm) was recorded after 6 hours using plate reader.

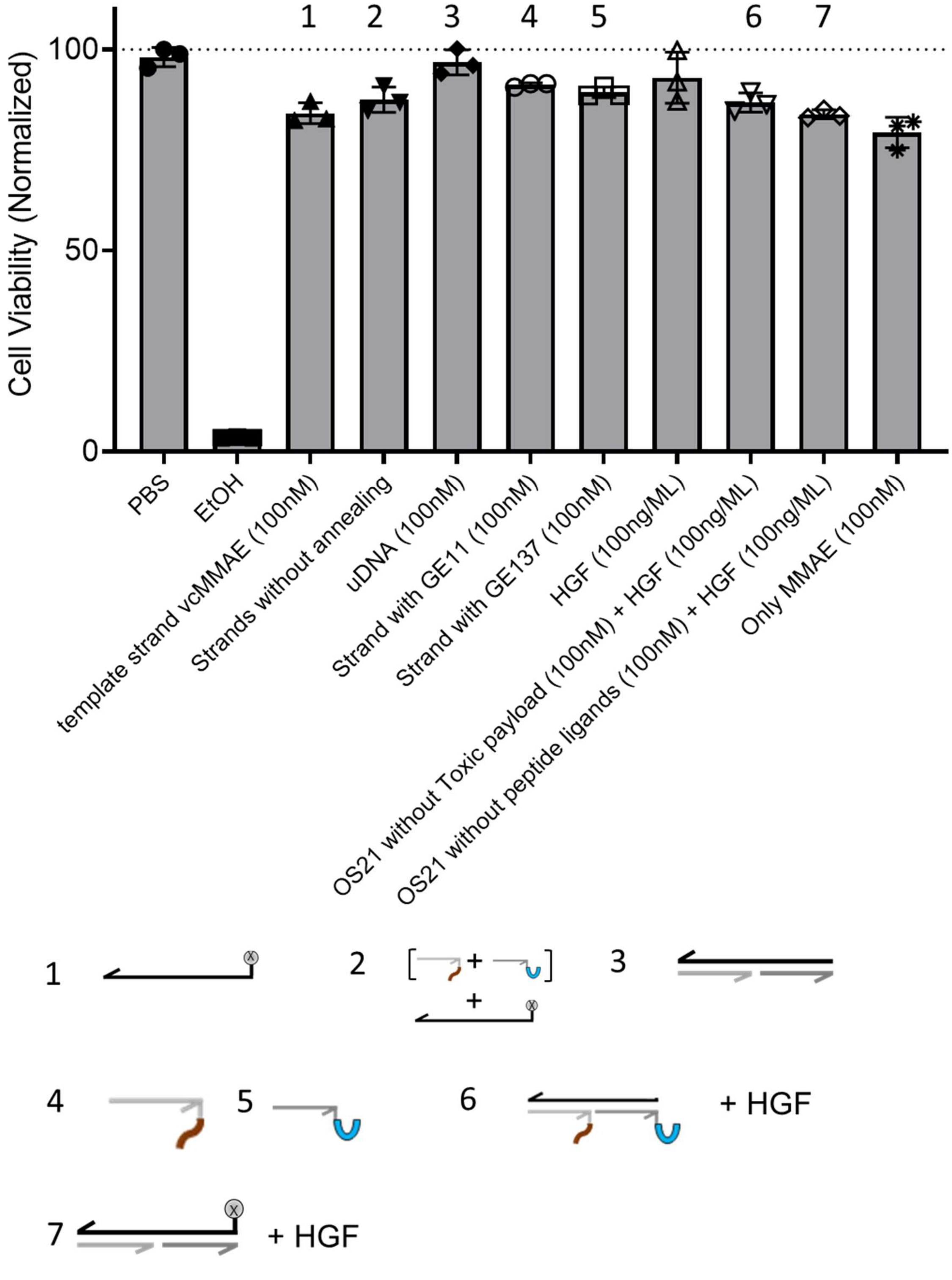

Fig. S17. Cytotoxicity of cathepsin B-cleavable vcMMAE-DNA-peptide complex with different controls, conditions: 10^4 cells/well in 100 µL medium and propagated 24 h to a confluency of ca. 85% cells, probes were incubated for 10 min in PBS, 37°C, 5% CO_2; incubation 10% Alamar blue in respective full medium, 6 h.

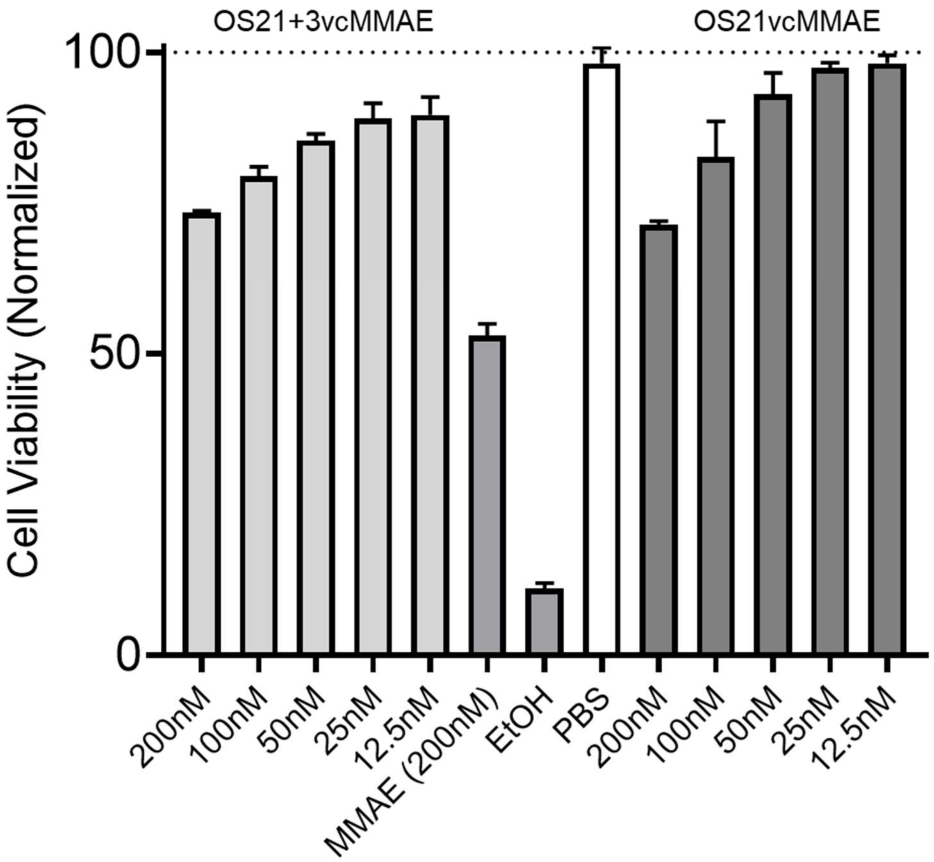

Fig. S18 Cytotoxicity of cathepsin B-cleavable vcMMAE-DNA-peptide complex with CHO cells, conditions: 10^4 cells/well in 100 µL medium and propagated 24 h to a confluency of ca. 85% cells, probes were incubated for 10 min in PBS, 37°C, 5% CO_2; incubation 10% Alamar blue in respective full medium, 6 h.

Table S2. EC50 calculation for OS21+3_vcMMAE in GraphPad prism 8.

[Inhibitor] vs. response (three parameters)	
Model used for the calculation	
Best-fit values	
Bottom	12,01
Top	104,6
IC50	9,146
logIC50	0,9612
Span	92,59
95% CI (profile likelihood)	
Bottom	7,926 to 15,76
Top	94,30 to 118,5
IC50	6,219 to 13,16
logIC50	0,7937 to 1,119
Goodness of Fit	
Degrees of Freedom	18
R squared	0,9810
Sum of Squares	212,7
Sy.x	3,437
Constraints	
IC50	IC50 > 0
Number of points	
# of X values	21
# Y values analyzed	21

Table S3. EC50 calculation for OS21_vcMMAE in GraphPad prism 8.

[Inhibitor] vs. response (three parameters) Model used for the calculation	
Best-fit values	
Bottom	9,522
Top	93,50
IC50	26,77
logIC50	1,428
Span	83,98
95% CI (profile likelihood)	
Bottom	3,341 to 14,81
Top	88,54 to 99,18
IC50	19,47 to 36,93
logIC50	1,289 to 1,567
Goodness of Fit	
Degrees of Freedom	18
R squared	0,9852
Sum of Squares	171,2
Sy.x	3,084
Constraints	
IC50	IC50 > 0
Number of points	
# of X values	21
# Y values analyzed	21

References

1. P. Ghosh, H. T. Dinh, A. Kocak, A. Homer, P. Bou-Dip, S. Schlicht, O. Seitz. Bispecific DNA-Peptide Probes for Targeting Receptor Pairs on Live Cells. ChemRxiv. 2025;

doi:10.26434/chemrxiv-2025-qrhkn-v2, just accepted in Angew. Chem. Int. Ed. 2025, DOI: 10.1002/anie.202514237.
2. Given the transformed nature of the NSCLC cell lines, some fluctuation in expression over time must be expected, for further reading: S. W. Jarantow, B. S. Bushey, J. R. Pardinas, K. Boakye, E. R. Lacy, R. Sanders, M.l A. Sepulveda, S. L. Moores, M. L. Chiu, Impact of Cell-surface Antigen Expression on Target Engagement and Function of an Epidermal Growth Factor Receptor × c-MET Bispecific Antibody, J. Biol. Chem., 2015, 290, 24689-24704, DOI: https://doi.org/10.1074/jbc.M115.651653.
